# Structural basis of aggregative adherence fimbriae II interactions with sialic acid, mucin, and human intestinal cells

**DOI:** 10.1128/iai.00483-24

**Published:** 2025-03-03

**Authors:** Luke W. Hagin, Inácio Mandomando, Fernando Ruiz-Perez, Nathan T. Wright, Laura A. Gonyar

**Affiliations:** 1 Department of Pediatrics, University of Virginia 482305 , Charlottesville, Virginia, USA; 2 Centro de Investigação em Saúde de Manhiça, Maputo, Mozambique; 3 Instituto Nacional de Saúde (INS) 586683 , Maputo, Mozambique; 4 ISGLOBAL-Hospital Clínic, Universitat de Barcelona 16724 , Barcelona, Spain; 5 Department of Chemistry and Biochemistry, James Madison University 427465 , Harrisonburg, Virginia, USA; University of California San Diego, La Jolla, California, USA

**Keywords:** fibronectin, *Escherichia coli*, fimbriae, mucin, sialic acid, intestinal cells, biofilms, structural biology

## Abstract

Enteroaggregative *Escherichia coli* (EAEC) is a common cause of diarrhea worldwide and is associated with growth faltering in developing countries. EAEC are defined by a characteristic adherence pattern mediated by the aggregative adherence fimbriae (AAFs). Despite the critical role of AAF in the definition of the EAEC pathotype, it is not known what host molecules mediate adherence and EAEC pathogenesis during infection of the human gastrointestinal tract. Multiple receptor candidates have been proposed based on *in vitro* experimentation. We propose that AAFs interact with multiple receptors during colonization of the human gastrointestinal mucosa, and we hypothesize that structural features of the AafA protein (the major subunit of AAF variant II produced by EAEC strain 042) promote these diverse interactions. In this study, we utilize a panel of AafA variants encoding single amino acid substitutions to understand the role of individual residues in biofilm formation as well as adherence to mucin, fibronectin, and human intestinal cells. We identify both charged and uncharged residues that participate in these interactions, and these residues cluster in two regions of the protein that may define a binding pocket at the junction of polymerized subunits. Although both bovine submaxillary mucin and human fibronectin are sialylated molecules, adherence to mucin is diminished by the removal of sialic acid residues while adherence to fibronectin is not, suggesting that the mechanisms of adherence to these molecules are distinct. Overall, our data provide insight into the structural features that determine AAF/II binding to mucin, sialic acid, and human intestinal cells.

## INTRODUCTION

Enteroaggregative *Escherichia coli* (EAEC) is a common cause of diarrhea in the United States (1, 2) and the second most common cause of traveler’s diarrhea (2, 3). In 2011, a Shiga toxin-producing EAEC of serotype O104:H4 caused an international foodborne outbreak, resulting in 3,816 cases of gastroenteritis, 845 cases of hemolytic uremic syndrome (HUS), and 54 deaths (4–6). EAEC also causes diarrhea in developing countries (2, 7), with most children experiencing at least one episode by 2 years of age (8). Both symptomatic and asymptomatic EAEC infection correlates with growth faltering in children in developing settings (8–10). WHO estimated in 2021 that 149 million children worldwide experienced growth faltering, which leads to life-long deficits in growth, cognitive abilities, and socioeconomic potential. Inflammation is generally associated with growth faltering (11–13), but how EAEC contributes is obscure.

EAEC was first identified by a distinct adherence pattern to HEp-2 epithelial cells found to be mediated by the aggregative adherence fimbriae (AAFs) (14, 15), which are required for attachment to intestinal cells and the gastrointestinal mucosa. EAEC virulence factors are encoded on a virulence plasmid (pAA) and chromosomal islands, and expression of many of these factors, including AAF, is controlled by the transcriptional activator AggR (16, 17). EAEC strains express one of five related AAF variants designated AAF/I–V (15, 18–22). AAFs are chaperone-usher fimbriae related to the Dr family of adhesins (23). The structures of major and minor subunits of AAF types I, II, IV, and V have been reported (24–27). In a recent study that evaluated the prevalence of each AAF-type amongst EAEC strains collected in the Global Enteric Multi-Center Study across three geographical sites (Bangladesh, Mali, and Mozambique), AAF/I and AAF/IV were found to be the most common (23% and 22% of strains, respectively), with AAF/V (16%), AAF/III (9%), and AAF/II (8%) being less prevalent (28). AAF/II is produced by EAEC strain 042, which causes diarrhea in human volunteers (29). We have shown that AAF/II determines both the abundance and location (within the mucus layer) of EAEC 042 adherence to colonoids, an updated model system of the human gastrointestinal mucosa (30).

Multiple putative receptors for AAF have been identified using *in vitro* assays (31–34), but the roles of each of these molecules in natural interactions with the host are unknown. AAFs bind *in vitro* to the extracellular matrix components fibronectin and laminin, as well as cytokeratin 8 (24, 31). These molecules are not expressed on the apical surface of the mucosa in normal tissues and are therefore not likely to be accessible for bacterial binding during early infection. It is possible that these receptors are revealed upon tissue damage. Similarly, epidermal growth factor receptor (EGFR) has also been proposed as a candidate receptor for EAEC (34) and contributes to EAEC-induced IL-8 production in immortalized intestinal epithelial cells (35, 36). EGFR is generally restricted to the basolateral surface in normal tissue, but apical expression has been reported in some systems (37, 38).

Secreted and transmembrane mucins are highly abundant in the human colon and have been identified as putative receptors for AAF (32, 33). Mucins are highly glycosylated, and the addition of sialic acid and sulfates confer negative charges, which could contribute to electrostatic interactions with AAF (which all have predicted isoelectric points of approximately 9 [24]). AAFs have been shown to bind to charged heparan sulfate proteoglycans (32), but whether these molecules are expressed in the colonic mucosa is less clear (39). The secreted mucin MUC2 and the signaling transmembrane mucin MUC1 have both been identified as candidate AAF receptors (32, 33). While expressed in healthy human colon (40), MUC1 expression is greatly increased by AAF in human intestinal xenografts (33) and by EAEC infection in intestinal organoids (32). Expression of MUC1 in HEK293 cells enhanced adherence of EAEC expressing AAF types I, II, and III, suggesting that this mechanism of adherence may be conserved, and AAF/II expression in EAEC strain 042 was required for MUC1-dependent adherence (33). MUC1 was also required for EAEC-induced neutrophil migration through polarized T84 monolayers (33).

We speculate that AAFs bind multiple receptors during colonization of the human gastrointestinal mucosa, and we hypothesize that properties of the AafA protein determine these distinct interactions. In this study, we employ a panel of AafA variants encoding single amino acid substitutions to dissect the role of amino acid identity, charge, and putative receptor binding regions in biofilm formation as well as adherence to mucin, fibronectin, and human intestinal cells.

## MATERIALS AND METHODS

### Bacterial strains and growth conditions

The bacterial strains used in this study are described in Table S1. Before addition to colonoid cultures, bacterial strains were incubated for 16–18 hours at 37°C in Dulbecco’s modified Eagle’s medium (DMEM) with 0.4% glucose (Gibco) under static conditions. Cultures were then diluted to prepare an inoculum of approximately 2 × 10^6^ CFU/10 μl or 2 × 10^7^ CFU/100 μl, depending on the assay. The *aafA* gene encoding single amino acid changes was cloned into pBAD30 and expressed in EAEC 042*aafA*, as previously described (24). All strains were sequenced to confirm that only one amino acid in AafA was modified.

### Western blot analyses

Bacterial strains were grown statically for 20 hours in DMEM-high glucose with 2% arabinose and 100 μg/mL carbenicillin. Cultures were standardized by OD_600_ before Western blot analyses. For whole-cell lysates, cells were collected by centrifugation, washed, and lysed under denaturing conditions. Samples were probed with rabbit polyclonal AafA antisera (24) or anti-DnaK (Thermo Fisher) and visualized by chemiluminescence (BioRad). Densitometry was performed by Fiji ImageJ software to quantitate protein amount.

### Quantification of biofilm formation

The biofilm assay was performed as described (41) with modifications. Briefly, bacterial strains were grown to an OD_600_ of 1.0 in LB at 37°C with shaking and then diluted 1:50 into DMEM high glucose with 2% arabinose and 100 μg/mL carbenicillin in 48-well polystyrene plates (Corning). After 18–20 hours of incubation at 37°C under static conditions, plates were washed three times with phosphate-buffered saline (PBS) and fixed with 75% ethanol. The fixed biofilms were dried and stained with 0.5% crystal violet (Sigma). Biofilms were washed four times with PBS after staining and solubilized in 95% ethanol. The absorbance was determined at 570 nm.

### *In vitro* binding assay

To assess bacterial binding to bovine submaxillary mucin (Sigma) or human fibronectin (Corning), 96-well polystyrene plates (Corning) were coated overnight at 4°C with 25 μg/mL protein suspended in 100 mM Tris-HCl, pH 8.0. The next day, plates were washed three times with PBS and blocked with 3% BSA in PBS for 1 hour at 37°C. After blocking, plates were washed three times, and 100 μL overnight bacterial culture (adjusted to contain approximately 1 × 10^7^ CFUs) in DMEM-high glucose (Gibco) was added for 90 minutes at 37°C. Plates were then washed three times with PBS and treated with 0.1% Triton X-100/PBS, and adherent bacteria were enumerated by dilution and plating on Luria agar.

### Adherence to Caco-2 cells

Caco-2 cells (ATCC) were cultured as previously described (42). Briefly, 1 × 10^5^ cells were seeded per well of polystyrene 96-well plates (Corning). Because of the high cell density, monolayers were confluent by 1 day post-seeding and were infected 3–4 days post-seeding. Monolayers were washed three times with DMEM-high glucose without FBS or antibiotics prior to infection and then rested for 1 hour. Bacterial strains were added as indicated and incubated for 3 hours. The monolayers were then washed three times with PBS, lysed with 1% Triton X-100/PBS, and adherent bacteria were enumerated by dilution and plating on Luria agar.

### Adherence to human colonoids

Colonoid cultures were established from deidentified biopsy specimens from healthy subjects obtained after endoscopic or surgical procedures using previously described methods (43). Subjects provided informed consent at Johns Hopkins University and all methods were carried out in accordance with approved guidelines and regulations. All experimental protocols were approved by the Johns Hopkins University Institutional Review Board (IRB) (Protocol NA_00038329).

All cell culture media were prepared as reported previously (44). Advanced DMEM–F-12 medium supplemented with 1 × GlutaMAX (Gibco), 10 mM HEPES (Sigma-Aldrich), and 100 Units/mL penicillin-streptomycin (Sigma-Aldrich) was used as the basal medium. Complete medium with growth factor (CMGF+) is basal medium supplemented with 50% (vol/vol) Wnt3a-conditioned medium, 20% (vol/vol) R-spondin-1-conditioned medium, 10% (vol/vol) Noggin-conditioned medium, 1 × B27 supplement (Gibco), 1  mM *N*-acetylcysteine (Sigma), 1 × Primocin (InvivoGen), 50 ng/mL human epidermal growth factor (R&D Systems), 10 nM [Leu-15]-gastrin (AnaSpec), 500 nM A83-01 (Tocris), and 10 µM SB202190 (Tocris). Differentiation medium is comprised of basal medium (no penicillin-streptomycin added), 10% (vol/vol) Noggin-conditioned medium, 1 mM *N*-acetylcysteine (Sigma), 50 ng/mL human epidermal growth factor (R&D Systems), 10  nM [Leu-15]-gastrin (AnaSpec), 500  nM A83-01 (Tocris), and 10% fetal bovine serum (Sigma Aldrich).

Organoids were cultured as 3D cysts embedded in Matrigel (Corning) and passaged approximately every 7 days. 3D organoids were harvested by gentle scraping in TrypLE express (Gibco) supplemented with 10 µM Y-27632 (Tocris), incubated at 37°C for 4 minutes, triturated approximately 30 times, washed using an equal volume of basal medium, and collected by centrifugation at 500 × *g* for 5 minutes. For passaging, the pellet was resuspended in Matrigel and seeded such that each well contained at least 50 organoids. The plate was incubated at 37°C for 10 minutes to allow the Matrigel to polymerize. A total of 0.5 mL of CMGF^+^ containing 10 µM each Y-27632 and CHIR99021 (Tocris) was added to each well. The medium was replaced with CMGF^+^ without Y-27632 and CHIR99021 after 48–72 hours. Fresh CMGF^+^ was added to the wells every other day.

To form monolayers, the triturated organoids were resuspended in CMGF^+^ containing Y-27632 and CHIR99021, as described previously (45, 46). Polystyrene 96-well plates (Corning) were precoated with 100  µL of a 34 µg/mL human collagen IV solution (Sigma) and incubated at 4°C overnight. 100 µL of resuspended organoid fragments was added to each well. Cultures were incubated at 37°C with 5% CO_2_. Monolayer confluence was assessed visually; typically, monolayer confluence was achieved in 7–10  days. Confluent monolayers were differentiated by incubation with Wnt3A-free and R-spondin-1-free medium (differentiation medium) for 3–5 days. Prior to infection of colonoid monolayers, the medium was changed and cells rested for 1 hour before infection. Bacterial strains were added as indicated and incubated for 3 hours. The monolayers were then washed three times with PBS, lysed with 1% Triton X-100/PBS, and adherent bacteria were enumerated by dilution and plating on Luria agar.

### Treatment with neuraminidase

To test the role of sialic acid in EAEC adherence, cells or substrates were pretreated with α2–3,6,8,9 neuraminidase A (NA, NEB), a broad-spectrum sialidase that will remove linear and branched terminal sialic acid residues from glycoproteins and oligosaccharides. NA was heat-inactivated by incubation at 65°C for 10 minutes. For *in vitro* binding assays, mucin or fibronectin was incubated with 25U active or heat-inactivated NA for 2 hours, and then the indicated substrate was diluted to 25 μg/mL protein suspended in 100 mM Tris-HCl, pH 8.0 for overnight coating of wells in a 96-well plate. For adherence assays with Caco-2 cells, monolayers were washed three times with DMEM-high glucose without FBS or antibiotics and then incubated with NA (25 or 50U) for 1 hour. Monolayers were then washed once with DMEM, and bacterial strains were added as indicated.

### Modeling of AafA structure

We created a model of two AafA subunits assembled as a dimer using the Alpha Fold 2 protocol found at ColabFold v1.5.5: AlphaFold2 using MMseqs2 (47, 48). A previously defined structure of AafA as a monomer was used as a template (PDB: 2MPV) (24), which was generated using donor strand complementation. We constructed novel hypothetical molecules that enabled donor strand complementation of two monomers assembled into a dimer (Fig. S4). PyMOL was used to visualize the dimer.

### Statistics

Statistical analyses were performed using GraphPad Prism (GraphPad Software, La Jolla, California). For most experiments, data were analyzed by one-way ANOVA followed by Bonferroni’s test for multiple comparisons. For data generated from adherence assays, log-transformed data were analyzed by one-way or two-way ANOVA followed by Bonferroni’s test for multiple comparisons.

## RESULTS

### Initial characterization of AafA variants

**Fig 1:**
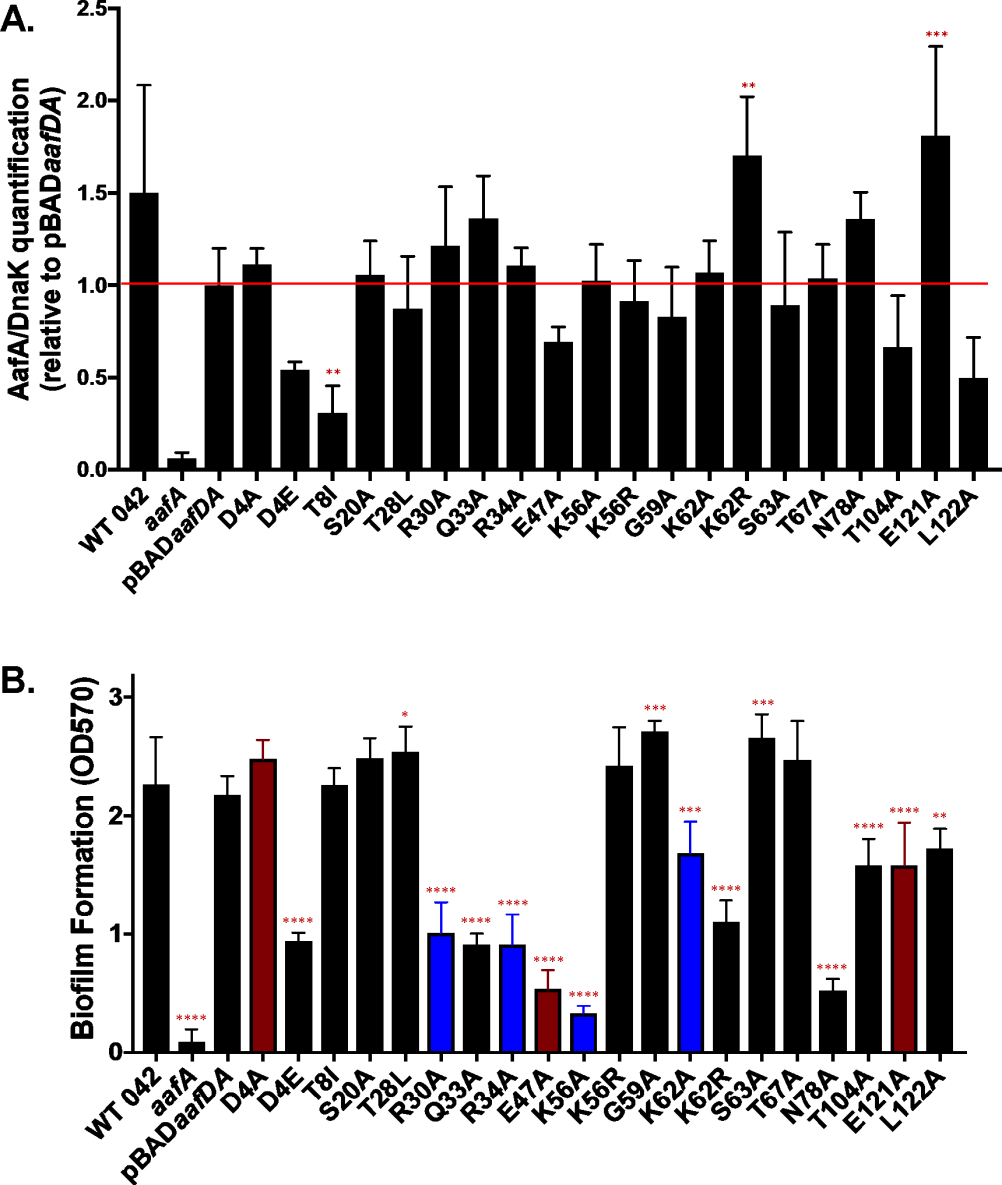
Quantification of AafA expression and biofilm formation in a panel of AafA variants with single amino acid substitutions. (A) Bacterial strains were grown statically for 20 hours in DMEM-high glucose with 2% arabinose and 100 μg/mL carbenicillin. Densitometry was performed with ImageJ on three immunoblots probed with anti-AafA and anti-DnaK antibodies, using DnaK as an endogenous control. One-way ANOVA was performed with Bonferroni’s test for multiple comparisons. Significance indicated as compared to 042*aafA*(pBAD*aafDA*). **P ≤ 0.01, ***P ≤ 0.001. (B) Bacterial strains were incubated for 18–20 hours at 37°C under static conditions in wells containing DMEM high glucose with 2% arabinose and 100 μg/mL carbenicillin. Plates were washed three times with PBS and fixed with 75% ethanol. The fixed biofilms were dried and stained with 0.5% crystal violet. Biofilms were washed four times with PBS after staining and solubilized in 95% ethanol. The absorbance was determined at 570 nm. Red indicates a change from a negatively charged residue (E47A and E121) and blue indicates a change from a positively charged residue (R30A, R34A, K56A, and K62A), all to neutrally charged alanine. One-way ANOVA was performed followed by Bonferroni’s test for multiple comparisons. Significance indicated as compared to 042*aafA*(pBAD*aafDA*). *P ≤ 0.05, **P ≤ 0.01, ***P ≤ 0.001, ****P ≤ 0.0001.

A panel of strains encoding *aafDA* in pBAD30 with sequence modifications resulting in a single amino acid substitution in AafA was constructed, as previously described (Table S1) (24). Amino acids selected for mutation are predicted to be surface exposed based on the published structure of AafA (24). Strains were grown in DMEM-high glucose containing 2% arabinose to induce the expression of *aafDA*. Expression and functionality of the 20 AafA variants were probed by western blot and biofilm analyses, respectively. Only one variant (T8I) had a significant reduction in AafA expression (normalized by the bacterial protein DnaK as an endogenous control) (Fig. 1A). Two variants (K62R and E121A) displayed a significant overexpression of AafA (Fig. 1A). To determine whether these statistically significant changes in expression level impacted function, the strains were grown statically in plastic wells for 20 hours, and biofilm formation was quantified by crystal violet staining. When comparing the protein expression data with the ability to form biofilm, the strain with the lowest AafA expression (T8I) formed biofilm at wild-type levels (Fig. 1). By contrast, the two strains with elevated AafA expression (K62R and E121A) both displayed significant reductions in biofilm formation (Fig. 1B). From these results, we speculate that the variation we observed in expression *in vitro* is not enough to affect function and that the level of expression induced by arabinose is sufficient to assess structure-function relationships.

Many of the substitutions that altered the charge at the indicated residue disrupted the ability to form biofilm at wild-type levels. This included two changes from a negatively charged residue (E47A and E121A) and four changes from a positively charged residue (R30A, R34A, K56A, and K62A), all to neutrally charged alanine (Fig. 1B). To further explore the role of charge in AAF/II-mediated biofilm formation, pairs of modifications at each of three specific residues were tested: one modification that conserved the charge by changing to another charged residue and one that neutralized the charge by changing to alanine. Aspartic acid at position 4 was changed to either glutamic acid (D4E) to conserve the negative charge or alanine (D4A) to disrupt the negative charge. At this residue, conserving the charge (D4E) decreased biofilm formation while neutralizing the charge (increasing the global positive charge of the protein) did not alter the amount of biofilm produced (Fig. 1B). Lysines at positions 56 and 62 were changed to arginine to conserve the positive charge or alanine to neutralize the charge at that residue. K56A, with a loss of positive charge, produced less biofilm; K56R, which conserved the positive charge at that residue, did not alter biofilm production (Fig. 1B). By contrast, both K62A and K62R formed less biofilm than the strain expressing the wild-type version of the protein (Fig. 1B). Modifying selected uncharged residues to alanine also impacted biofilm formation; Q33A, N78A, T104A, and L122A formed significantly less biofilm than the strain expressing wild-type AafA (Fig. 1B). These data implicate both charged and uncharged residues in AAF-mediated biofilm formation.

### *In vitro* adherence to mucin

**Fig 2:**
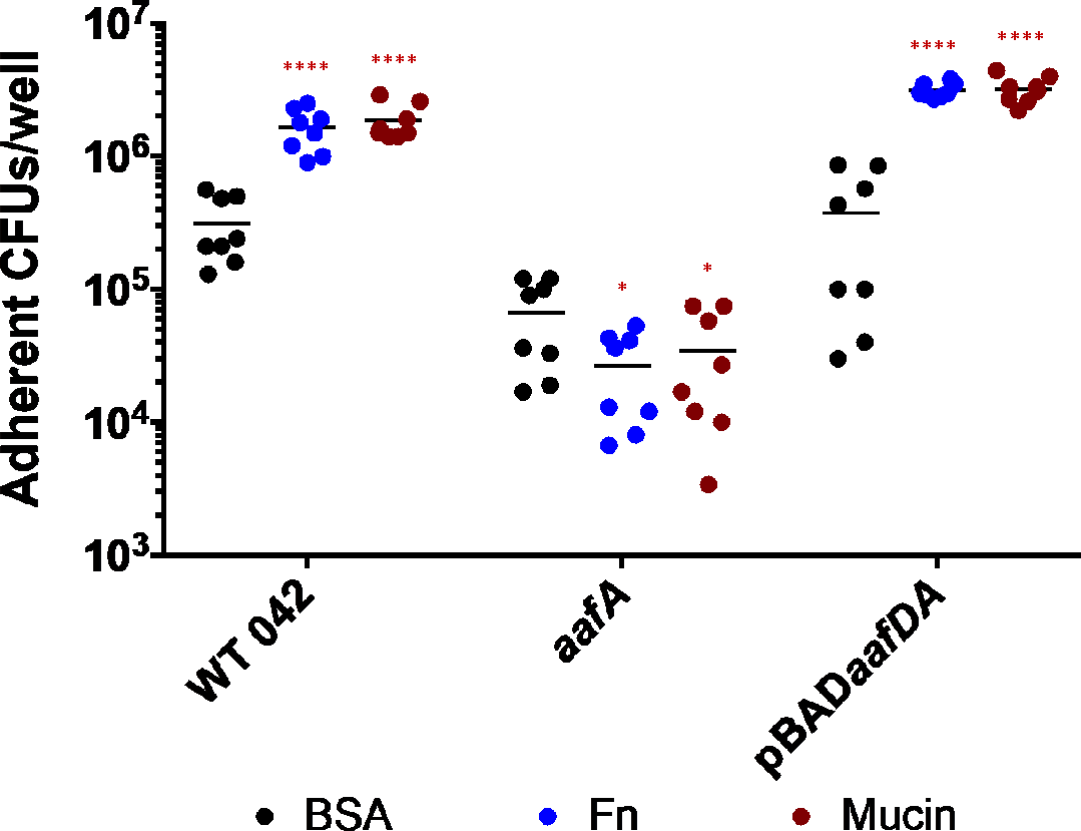
EAEC binds to mucin and fibronectin, and adherence to both substrates is AAF-dependent. Bacterial strains were incubated in polystyrene plates precoated with bovine submaxillary mucin or human fibronectin for 90 minutes at 37°C. Plates were then washed three times with PBS and treated with 0.1% Triton X-100/PBS, and adherent bacteria were enumerated by dilution and plating. Two-way ANOVA was performed on log-transformed data followed by Bonferroni’s test for multiple comparisons. Significance indicated as compared to BSA control. *P ≤ 0.05, ****P ≤ 0.0001.

To assess binding to mucin *in vitro*, we coated 96-well plates with bovine submaxillary mucin, blocked for non-specific binding with BSA, incubated with bacteria for 90 minutes, washed, and then quantified adherent bacteria by plating CFUs. Fibronectin was used as a positive control and BSA was used as a negative control. Binding to mucin was increased compared to BSA in strains expressing AAF/II (WT 042), *aafA* repair (where the wild-type sequence of *aafA* has been restored at the native location within the pAA2 virulence plasmid), and 042*aafa*(pBAD*aafDA*)) but not in a strain lacking AAF/II (042*aafa*) (Fig. 2), suggesting that EAEC adherence to bovine submaxillary mucin is AAF-dependent. Expression of AAF/II also correlated with increased binding to fibronectin, as previously described (Fig. 2).

**Fig 3:**
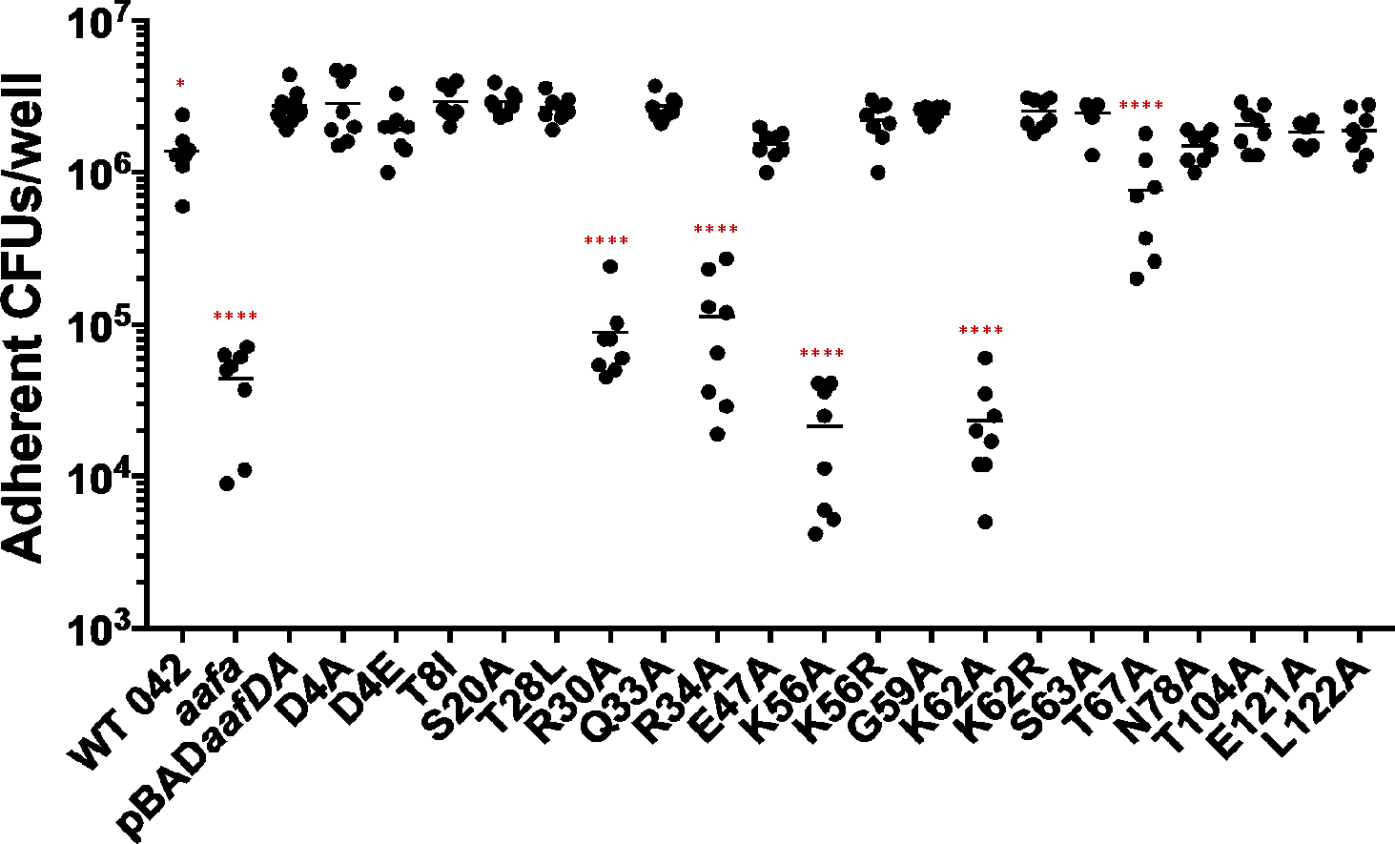
Identification of AafA residues that contribute to binding to bovine submaxillary mucin. Bacterial strains were incubated in polystyrene plates precoated with bovine submaxillary mucin for 90 minutes at 37°C. Plates were then washed three times with PBS and treated with 0.1% Triton X-100/PBS, and adherent bacteria were enumerated by dilution and plating. One-way ANOVA was performed on log-transformed data followed by Bonferroni’s test for multiple comparisons. Significance indicated as compared to 042*aafA*(pBAD*aafDA*). ****P ≤ 0.0001.

Next, we screened the panel of AafA variants to identify substitutions that decreased AAF-dependent binding to mucin. Bacterial strains were incubated with plates coated with bovine submaxillary mucin for 90 minutes, adherent bacteria were quantified by plating, and then the level of adherence was compared to 042*aafa*(pBAD*aafDA*) expressing the wild-type sequence of AafA. The largest defects were observed in the four mutations (R30A, R34A, K56A, and K62A) that resulted in a change from a positively charged residue to a neutrally charged alanine (Fig. 3). Conservation of the charge in substitutions at lysine residues (K56R and K62R) did not alter mucin binding (Fig. 3). A strain expressing a substitution at one additional residue, T67A, bound significantly less to mucin than the control strain expressing wild-type AafA (Fig. 3).

### Adherence to human intestinal cells

**Fig 4:**
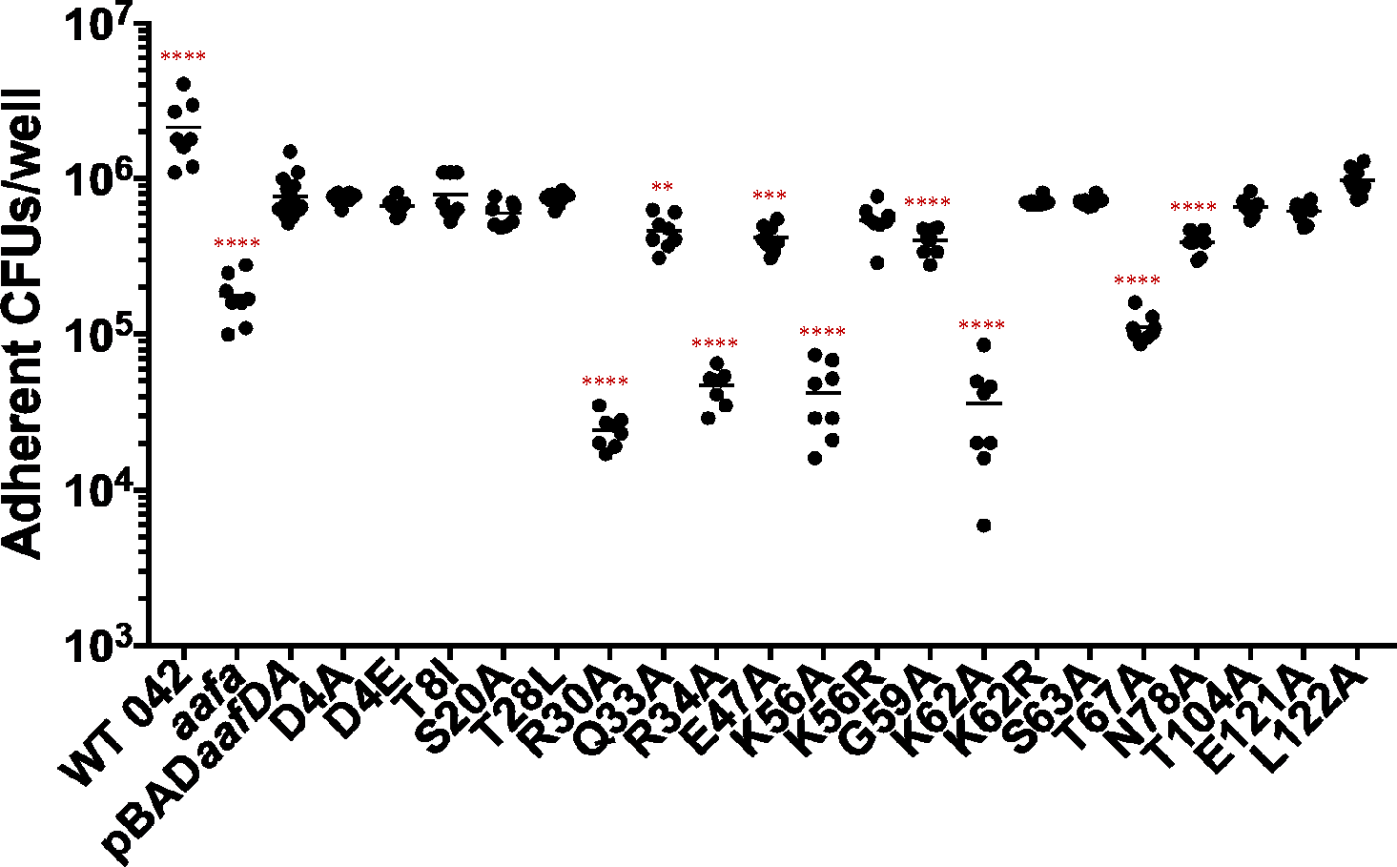
Identification of AafA residues that contribute to binding to Caco-2 cells. Bacterial strains were added to prewashed, confluent Caco-2 monolayers and incubated for 3 hours. The monolayers were then washed three times with PBS, lysed with 1% Triton X-100/PBS, and adherent bacteria were enumerated by dilution and plating. One-way ANOVA was performed on log-transformed data followed by Bonferroni’s test for multiple comparisons. Significance indicated as compared to 042*aafA*(pBAD*aafDA*). **P ≤ 0.01, ***P ≤ 0.001, ****P ≤ 0.0001.

Next, we probed the ability of AafA variants to bind to human intestinal cells. We employed two model systems: Caco-2 cells and human colonoids. Caco-2 cells are intestinal epithelial cells isolated from the colon tissue of a 72-year-old male with colorectal adenocarcinoma. Caco-2 cells were seeded in 96-well plates at a high density and were fully confluent at the time of bacterial infection. Caco-2 monolayers were infected with bacterial strains for 3 hours and then adherent bacteria were quantified by plating. Adherence of an *aafA*-deficient strain was significantly reduced compared to wild-type 042 and 042*aafa*(pBAD*aafDA*) expressing the wild-type sequence of AafA (Fig. 4), suggesting that AAF/II mediates adherence to Caco-2 cells. Next, we screened the panel of AafA variants. Consistent with the results from the mucin binding assay, the largest defects were observed in the four mutations (R30A, R34A, K56A, and K62A) that resulted in a loss of a positively charged residue (Fig. 4). Conservation of the charge in substitutions at lysine residues (K56R and K62R) did not impact the ability to bind to Caco-2 cells (Fig. 4). Additional substitutions also significantly reduced adherence to Caco-2 cells: Q33A, E47A, G59A, T67A, and N78A (Fig. 4). T67A, which also exhibited decreased binding to mucin, adhered to Caco-2 cells at significantly lower levels (*P* < 0.001) compared to Q33A, E47A, G59A, and N78A (Fig. 4).

**Fig 5:**
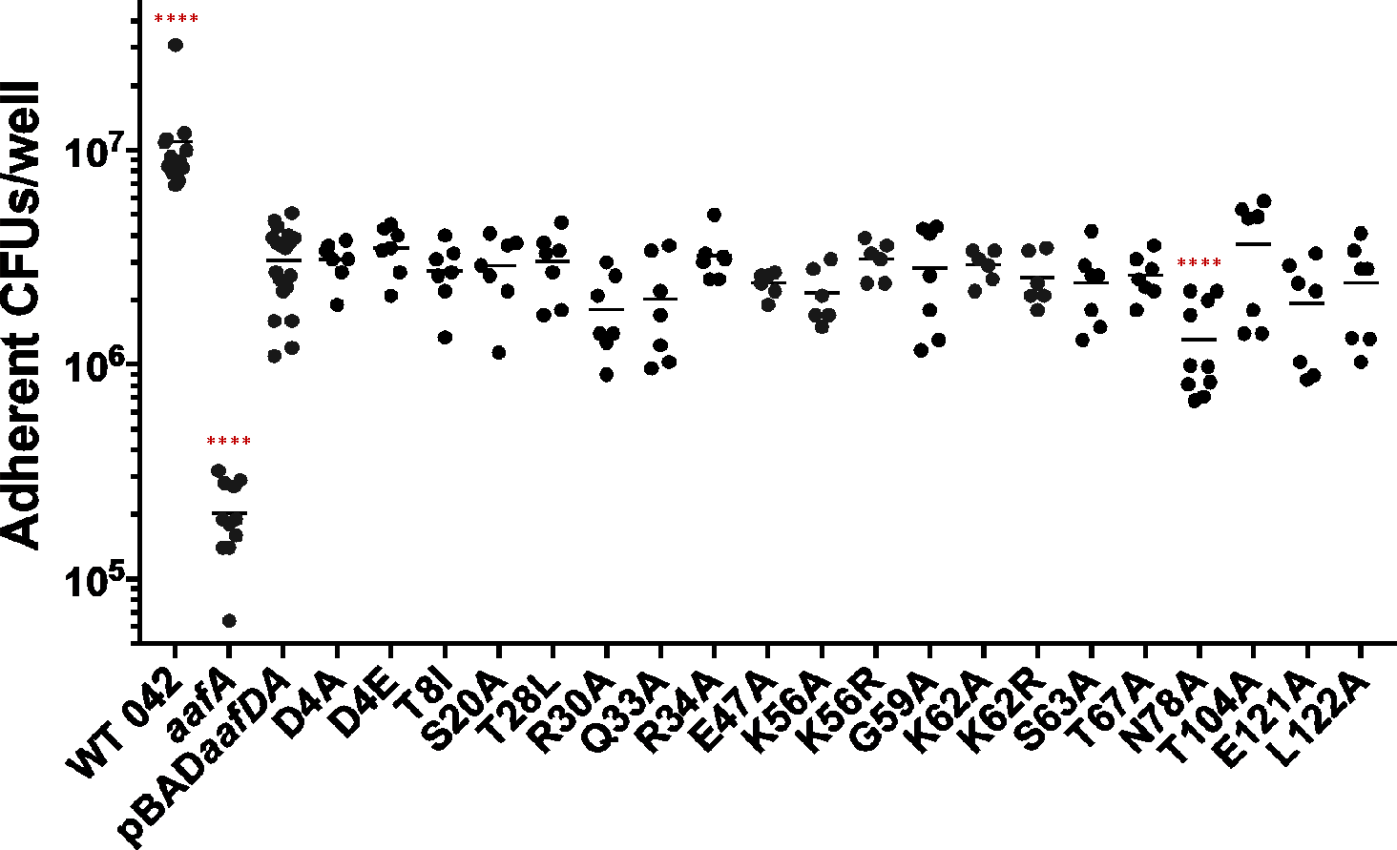
Identification of AafA residues that contribute to binding to human colonoids. Bacterial strains were added to prewashed, confluent colonoid monolayers (line 75C) and incubated for 3 hours. The monolayers were then washed three times with PBS, lysed with 1% Triton X-100/PBS, and adherent bacteria were enumerated by dilution and plating on Luria agar. One-way ANOVA was performed on log-transformed data followed by Bonferroni’s test for multiple comparisons. Significance indicated as compared to 042*aafA*(pBAD*aafDA*). ****P ≤ 0.0001.

We have previously established a role for AAF/II in determining both the abundance and location of EAEC adherence to human colonoids (30). Colonoid lines are derived from stem cells isolated from biopsies from healthy, consenting human donors. Confluent colonoid monolayers seeded in 96-well plates were differentiated as previously described, leading to segment-specific expression of host molecules and the production of a mucus layer (30, 45). Differentiated colonoid monolayers were infected with bacterial strains for 3 hours and then adherent bacteria were quantified by plating. Surprisingly, only one variant (N78A) adhered significantly less than 042*aafa*(pBAD*aafDA*) expressing the wild-type sequence of AafA (Fig. 5); this variant also adhered less to Caco-2 cells (Fig. 4). The four variants (R30A, R34A, K56A, and K62A) with a loss of positive charge adhered to colonoids at wild-type levels (Fig. 5), in contrast to the low level of adherence of these variants to bovine submaxillary mucin and Caco-2 cells. Another variant (T67A), with a modification at an uncharged residue, was defective in adherence to bovine submaxillary mucin and Caco-2 cells also adhered at wild-type levels (Fig. 5).

### Role of sialic acid in AAF-mediated adherence

**Fig 6:**
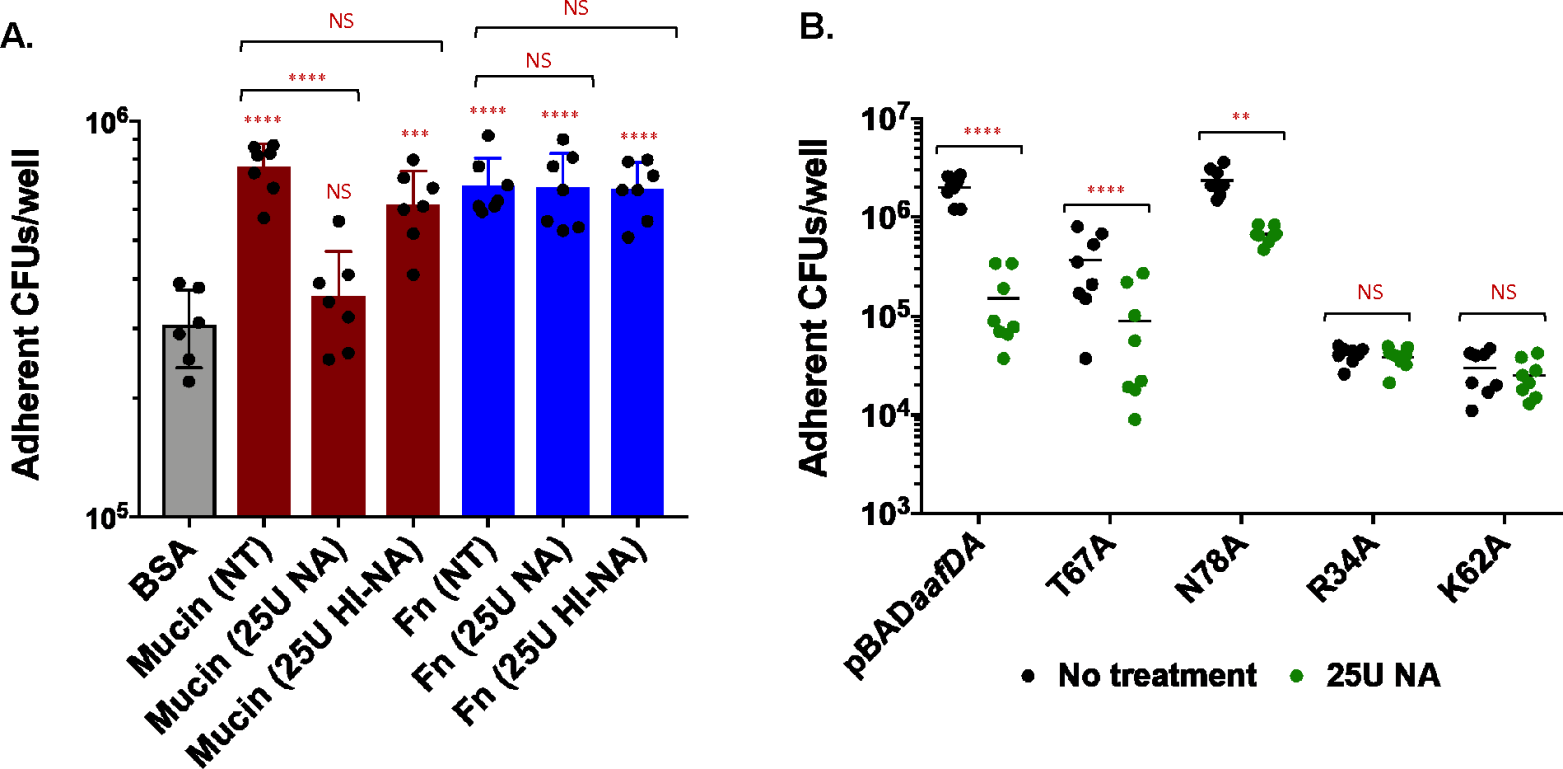
Role of sialic acid in EAEC adherence to mucin and fibronectin. Bovine submaxillary mucin or human fibronectin was incubated with 25U active or heat-inactivated NA for 2 hours, and then the indicated substrate was used to coat wells in a 96-well plate. (A) EAEC strain 042 was incubated in coated plates for 90 minutes at 37°C. Wells were then washed three times with PBS and treated with 0.1% Triton X-100/PBS, and adherent bacteria were enumerated by dilution and plating. One-way ANOVA was performed on log-transformed data followed by Bonferroni’s test for multiple comparisons. Significance is indicated as compared to BSA control unless indicated. **P ≤ 0.01, ****P ≤ 0.0001. (B) Bacterial strains were incubated in mucin-coated plates for 90 minutes at 37°C and then processed as already described. Two-way ANOVA was performed on log-transformed data followed by Bonferroni’s test for multiple comparisons. Comparisons as indicated. **P ≤ 0.01, ****P ≤ 0.0001.

The addition of sialic acid is a common post-translational modification of human mucus and confers a negative charge. Sialic acids comprise a family of acidic, nine carbon sugars that commonly reside at the terminal position of glycosylated molecules. Sialic acid modification of mucus is higher in the colon than in other intestinal segments (49, 50). Neuraminidase treatment, which cleaves sialic acid, reduces EAEC agglutination of red blood cells and EAEC adherence to human HEK293 cells transfected with MUC1, suggesting that the AAF/II receptor is sialylated (33). Based on these findings, we probed the role of sialic acid in adherence to fibronectin, mucin, and Caco-2 cells. Fibronectin and mucin were incubated with 25U of neuraminidase or heat-inactivated neuraminidase for 2 hours, and then binding to these substrates was assessed in 96-well plates. Both fibronectin and mucin are highly sialylated molecules (51, 52). Adherence of 042 to mucin was significantly reduced after neuraminidase treatment compared to untreated mucin, and treatment with heat-inactivated neuraminidase did not reduce 042 adherence to mucin (Fig. 6A). 042 adhered equivalently to untreated, neuraminidase-treated, and heat-inactivated neuraminidase-treated fibronectin (Fig. 6A). These data suggest that adherence to mucin involves a sialylated glycan but adherence to fibronectin does not.

Next, we investigated the role of sialic acid in the ability of variants in specific AafA residues to bind to mucin *in vitro*. We selected a charged and uncharged residue from each of the AafA regions implicated in binding to mucin: N78A, R34A, T67A, and K62A. Strains expressing WT AafA and AafA containing modifications to uncharged residues (N78A and T67A) adhere significantly less to neuraminidase-treated mucin compared to untreated mucin (Fig. 6B). Strains expressing AafA with substitutions in charged residues (R34A and K62A) adhere at levels equivalent to 042*aafA*, and this adherence was not sensitive to neuraminidase treatment (Fig. 6B), suggesting that these two variant fimbriae do not adhere to mucin regardless of sialic acid modification.

**Fig 7:**
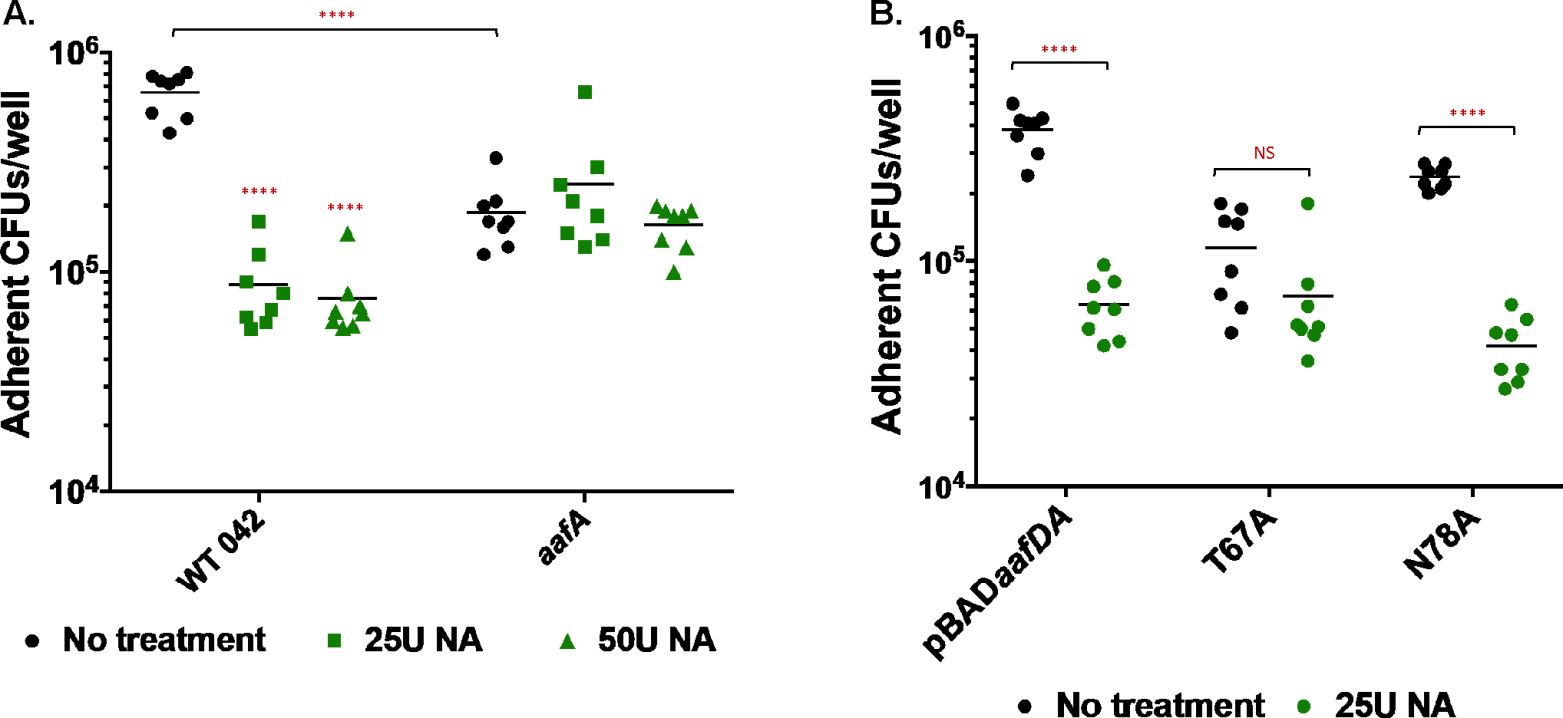
Role of sialic acid in EAEC adherence to Caco-2 cells. (A) Confluent Caco-2 monolayers were incubated with 25U or 50U NA for 1 hour, and then bacterial strains were added for 3 hours. Monolayers were then washed three times with PBS and lysed with 0.1% Triton X-100/PBS, and adherent bacteria were enumerated by dilution and plating. Two-way ANOVA was performed on log-transformed data followed by Bonferroni’s test for multiple comparisons. The comparison shown is to no treatment group unless indicated. ****P ≤ 0.0001. B. Confluent Caco-2 monolayers were incubated with 25U for 1 hour. Bacterial strains were added for 3 hours, and then processed as already described. Two-way ANOVA was performed on log-transformed data followed by Bonferroni’s test for multiple comparisons. Comparisons as indicated. ****P ≤ 0.0001.

Caco-2 cells were treated with 25U or 50U of neuraminidase for 1 hour before infection with EAEC strains. Neuraminidase treatment significantly reduced adherence of WT 042 to Caco-2 cells but did not reduce adherence of 042*aafA* (Fig. 7A), suggesting that AAF-dependent adherence to Caco-2 cells involves a sialylated receptor. To understand how specific residues impact sialic acid-dependent adherence to Caco-2 cells, we probed adherence of the T67A and N78A strains to Caco-2 cells with or without neuraminidase treatment (Fig. 7B). Adherence of strains expressing wild-type AafA or AafA with the N78A substitution was sensitive to neuraminidase treatment (Fig. 7B). Although there may be a trend toward decreased adherence of the strain expressing AafA T67A after neuraminidase treatment, this difference was not statistically significant, leading us to conclude that the T67A substitution decreased sensitivity to neuraminidase treatment under our experimental conditions (Fig. 7B).

### Structural analysis of mutated residues in AafA

Four of the AafA variants studied (R30A, R34A, K56A, and K62A) were defective in biofilm formation, adherence to mucin, and adherence to Caco-2 cells (Table 1). Interestingly, all four of these variant strains adhered to colonoids at wild-type levels and significantly higher levels than 042*aafA* (Fig. 5). We detected equivalent expression of AafA protein by Western blot analyses (Fig. 1A), and a previous study used qualitative immunofluorescence to suggest that these four variants express AafA extracellularly (24). To better understand how selected substitutions (R30A, R34A, K56A, K62A, T67A, and N78A) alter AafA structure, we utilized AlphaFold (47) to model protein folding and compared variant structures to wild-type AafA. Four of the variants (R30A, K62A, N78A, and T67A) were highly similar to the wild type (Fig. S1). AlphaFold predictions of structures containing R34A and K56A substitutions exhibited more uncertainty (represented by regions of warmer color in Fig. S1). Despite the slight uncertainty of structure prediction by AlphaFold for K56A, we were able to detect the presence of structures indistinguishable from native AAF/II by transmission electron microscopy (Fig. S2). In images of the R34A variant, we observed structures distinct from flagella and of a similar size and diameter to native AAF/II; however, these structures did not form bundles characteristic of AAF/II and had a more jagged appearance (Fig. S2). As these structures were lacking in 042*aafA*, we speculate that they are likely AAF and that the R34A mutation may mediate these minor morphological differences. These data, in combination with the ability to adhere to colonoids at significantly higher levels than an *aafA*-deficient mutant, suggest that fimbrial structures are assembled in the K56A and R34A. Therefore, phenotypic changes due to single amino acid modifications are likely due to perturbations in structure versus a complete lack of fimbrial structures.

**TABLE 1:** Summary of adherence phenotypes associated with AafA variants^*a*^

Strain	Fn (from (24))	Mucin	Biofilm formation	Caco-2	Colonoid
**D4A**	**+**	**+**	**+**	**+**	**+**
**D4E**	**Not tested**	**+**	**-**	**+**	**+**
**T8I**	**+**	**+**	**+**	**+**	**+**
**S20A**	**+**	**+**	**+**	**+**	**+**
**T28L**	**+**	**+**	**+**	**+**	**+**
**R30A**	**-**	**-**	**-**	**-**	**+**
**Q33A**	**+**	**+**	**-**	**+**	**+**
**R34A**	**-**	**-**	**-**	**-**	**+**
**E47A**	**+**	**+**	**-**	**+**	**+**
**K56A**	**-**	**-**	**-**	**-**	**+**
**K56R**	**+**	**+**	**+**	**+**	**+**
**G59A**	**+**	**+**	**+**	**+**	**+**
**K62A**	**-**	**-**	**+**	**-**	**+**
**K62R**	**+**	**+**	**+**	**+**	**+**
**S63A**	**+**	**+**	**+**	**+**	**+**
**T67A**	**+**	**-**	**+**	**-**	**+**
**N78A**	**+**	**+**	**-**	**-**	**-**
**T104A**	**+**	**+**	**+**	**+**	**+**
**E121A**	**+**	**+**	**+**	**+**	**+**
**L122A**	**+**	**+**	**+**	**+**	**+**

^*a*^ Although smaller differences were determined to be statistically significant as indicated in figures, only the greatest defects are shown in this table using a threshold of less than half the control strain expressing wild-type AafA. A reduction in adherence or biofilm formation compared to a strain expressing wild-type AafA is indicated by (-); variants that are equivalent to or greater than wild-type are indicated by (+).

**Fig 8:**
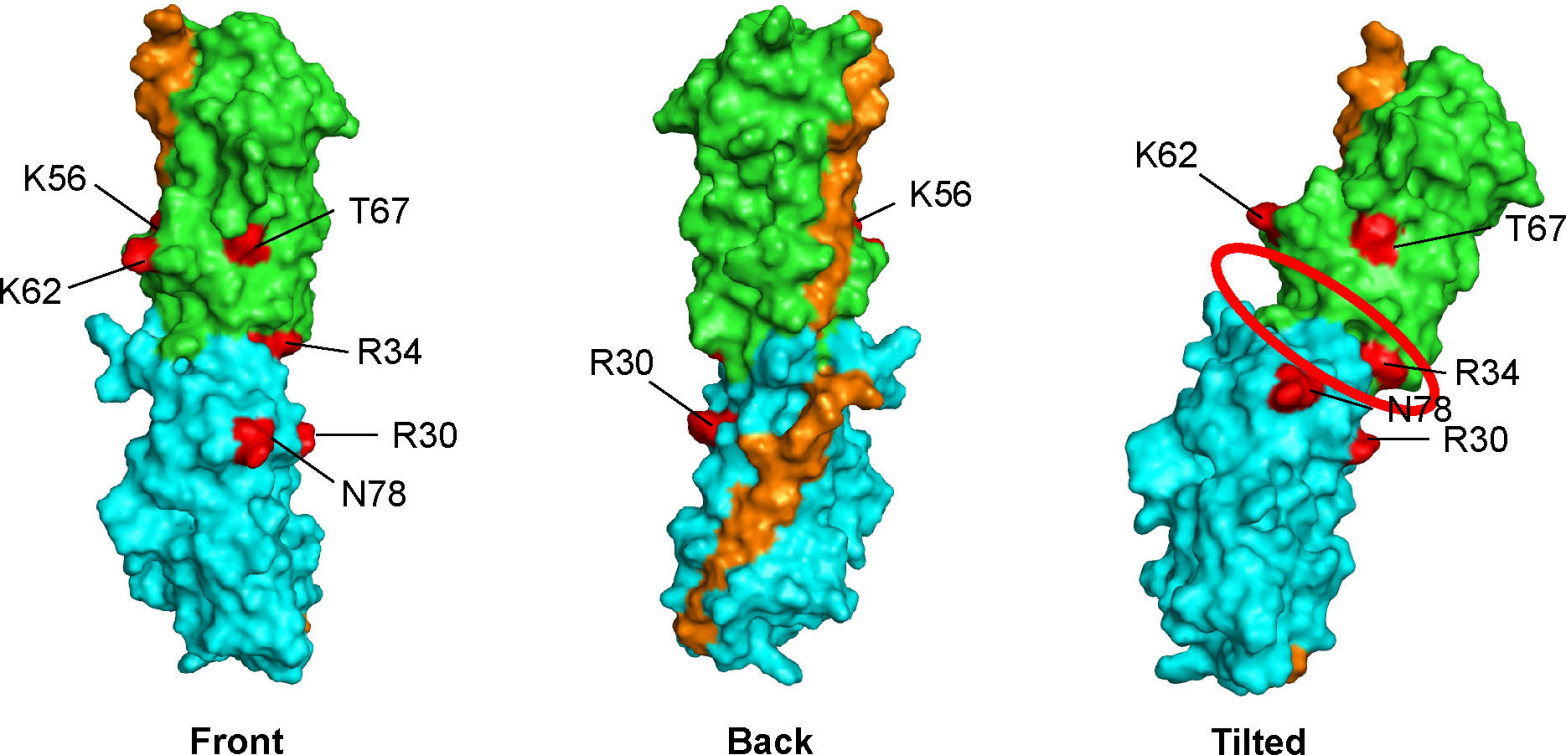
Model of AafA dimer. Images generated in PyMOL. Residues implicated in binding to Caco-2 cells are colored in red. Molecule 1 (green), molecule 2 (blue), and n-terminal extensions (orange) are indicated. Three views of the molecule (front, back, and tilted) are shown. Proposed binding pocket in groove between subunits indicated with a red circle in tilted view.

We mapped AafA residues implicated in adherence in our assays onto the AafA monomeric structure (PDB: 2MPV) to ascertain whether they compromise a binding pocket (Fig. 8). K56A, K62A, and T67A localized to one region of the protein, and R30A, R34A, and N78A localized to a separate region of the protein (Fig. 8). We hypothesized that these six residues could be juxtaposed to form a receptor-binding pocket when AafA was polymerized into the AAF filament. To visualize how these two regions are orientated in a polymerized fimbria, we assembled a predicted AafA dimer using the Alpha Fold 2 protocol found at ColabFold v1.5.5 (24, 47, 48). In this model, we removed the native N-terminal sequence corresponding to the donor strand complementation sequence of one subunit and duplicated this same sequence at the C-terminus of the other subunit (Fig. S3). Each monomer in the resulting model is similar to the previously solved NMR structure, and the resulting model contains the expected donor strand complementation, modeled with a high degree of confidence (Fig. 8). The two monomers adjoined with a rotation approximating 77°. The dimer interface forms a groove similar to that predicted for the AggA (AAF/I) filament (24) (Fig. 8).

We mapped the six residues that abrogated Caco-2 binding on the dimer structure (Fig. 8) and found that they all localized to one face of the structure (indicated on the front view). Five of these six were predicted to lie in close proximity to the monomer interface groove shown in Fig. 8. We speculate that this region is important for receptor binding. Interestingly, the side of the structure opposite the six cell-binding residues included the two N-terminal extensions assembled into the folded structure.

Next, we used an existing data set (28) to determine whether the residues studied here are conserved amongst AAF/II-producing strains. Most residues were conserved (S20, T28, Q33, R34, E47, K56, G59, S63, T67, T104, E121, and L122). Even when the identity was not conserved, amino acids at a particular residue had similar properties and are considered a neutral change (D or E at position 4, R or K at position 30, and N or D at position 78). Residues that are not conserved include positions 8 (T or I) and 62 (K or A). Notably, strains encoding A62 have a substitution at residue 60 from G to K, conserving the positive charge in that region of the protein. Based on our findings that the N78A substitution decreased binding to intestinal cells, it would be interesting to investigate whether an N78D substitution altered binding to intestinal cells in future studies.

## DISCUSSION

AAF/II binds to the extracellular matrix components fibronectin, laminin, and type IV collagen (31). It has been demonstrated that binding to fibronectin by AAF/I and AAF/II involves a patch of positively charged residues, suggesting an electrostatic mechanism for binding (24). A subsequent study reported that AAF/V does not bind fibronectin, and AAF/V variants encode a conserved aspartic acid that disrupts a continuous band of positively charged residues hypothesized to be important for binding to fibronectin (25). Furthermore, when this aspartic acid was mutated to lysine, binding to fibronectin was increased (25). Together, these data support that binding to fibronectin, and potentially other extracellular matrix components, is largely electrostatic.

Although the binding of AAF to extracellular matrix components is conserved among many AAF types, the biological relevance of these interactions is unclear. In our study, we characterized the contribution of specific residues and regions in AafA to binding to mucin and two human intestine-derived cell lines. Mucins are high molecular weight glycoconjugates that are abundant in the human colon. Secreted, soluble mucins form a thick layer coating the colon epithelium and maintain separation from the resident microbial community, and EAEC must migrate through this protective layer to colonize the epithelial surface. The human colon also expresses transmembrane mucins that are likely involved in signaling and communication with the luminal environment to maintain host homeostasis. Soluble and secreted mucins have both been proposed as AAF receptors (32, 33). Here, we used bovine submaxillary mucin to screen for AAF-dependent EAEC binding to mucin. Bovine submaxillary mucin is a secreted mucin that is most similar to the human mucin MUC19, which is expressed in salivary glands and tracheal submucosal glands (53, 54). Importantly, bovine submaxillary mucin is highly sialylated (9%–24% bound sialic acid), consistent with the high levels of sialylation that have been reported for the human colonic mucin MUC2 (55, 56). EAEC binding to mucin was AAF-dependent (Fig. 2) and decreased by treatment with neuraminidase, implicating sialic acid modification in AAF-dependent EAEC binding to bovine submaxillary mucin (Fig. 6A). Both bovine submaxillary mucin and human fibronectin are highly sialylated (51, 52). Interestingly, neuraminidase treatment of fibronectin did not alter EAEC binding (Fig. 6A). These data highlight that AAF-mediated binding to mucin versus fibronectin may involve distinct regions or features of AAF.

We screened 20 strains expressing AafA with single amino acid substitutions and identified five variants that adhered to mucin significantly less than wild-type AafA (Fig. 3). Four of these variants (R30A, R34A, K56A, and K62A) involve charged residues previously implicated in electrostatic interactions with fibronectin (Table 1) (24). An additional variant, T67A, adhered to mucin less than wild type (Fig. 3) but was not defective for biofilm formation (Fig. 1) or binding to fibronectin (24). This residue is one of the few conserved residues among AAF types I, II, III, and V (25). Adherence to mucin by the T67A variant was still sensitive to neuraminidase treatment (Fig. 6B) but to a lesser extent than the wild type, suggesting that this variant retains binding to sialylated molecules but perhaps with less specificity (Fig. 6B).

Next, we screened the panel of AafA variants for binding to Caco-2 cells and human colonoids. Caco-2 cells are a simple, well-characterized model system of human intestinal epithelial cells. EAEC adherence to Caco-2 cells requires AAF and is inhibited by neuraminidase treatment, suggesting sialylation is involved in AAF-mediated EAEC adherence to Caco-2 cells. The residues implicated in binding to mucin (charged residues R30, R34, K56, and K62 as well as the uncharged residue T67) were also important for EAEC adherence to Caco-2 cells (Fig. 4). In addition, modest but significant reductions in adherence were observed for Q33A, E47A, G59A, and N78A (Fig. 4).

Caco-2 cells can be polarized when grown on Transwell inserts for 2 + weeks, but our experiments were not performed under those conditions. Even after polarization, Caco-2 cells do not form a mucus layer. Host molecules that are normally basolaterally restricted, like extracellular matrix components and EGFR, may be apically expressed in unpolarized monolayers and available for binding. However, we observed that binding to Caco-2 cells is neuraminidase sensitive while binding to fibronectin is not, which supports that these electrostatic interactions may not be the primary AAF-receptor interactions that promote binding to Caco-2 cells.

Despite great diversity in the ability of variants to form a biofilm, bind to mucin and/or fibronectin, and bind to Caco-2 cells, binding to human colonoids was relatively homogeneous. Only one variant, N78A, bound to colonoids significantly less than the wild type (Fig. 5). The N78A variant also adhered less to Caco-2 cells and formed less biofilm but was not defective in binding to mucin or fibronectin. The variants in charged residues, which exhibited the largest defects in most of our assays, adhered to colonoids at wild-type levels. These results may be explained by the increased complexity of this model system. Differentiated colonoids are polarized monolayers with segment-specific expression of surface molecules as well as the production of a mucus layer. Our results, in combination with previous reports, support that AAF can be associated with a variety of host molecules. Our working model is that these distinct AAF-receptor interactions may be spatial and temporal during the sequence of EAEC colonization of the human gastrointestinal mucosa. Although total EAEC adherence, as we quantified by enumerating the total bacterial cells adhered per monolayer, is equivalent among most of the variants screened, there could be qualitative differences in the way these variants are associated with the mucus layer and epithelial surface. Further dissection of these interactions is a natural next step to elucidate the contribution of AAF to EAEC migration through the mucus layer and colonization of the epithelial surface.

The majority of the substitutions utilized in this study were alanine, which is a common strategy for identifying the contribution of amino acid identity and specific side chains to protein function (57). Alanine is an uncharged, hydrophobic amino acid. Of note, all of the surface-exposed residues selected for mutation in this study are hydrophilic. It is possible that this change in hydrophobicity by substitution to alanine also contributes to changes in the AafA function. To address the role of hydrophobicity in future experiments, selected residues could be changed to glycine instead of alanine.

A model based on the solved structure of the AAF/I major subunit AggA identified a groove between polymerized subunits that were enriched in basic residues, and they suggest that this groove mediates binding to fibronectin (24). Using donor strand complementation, we generated a model of two AafA subunits polymerized together. We found that the six residues identified in our study localize together at the junction between the two subunits (Fig. 8) in a groove similar to that identified for AAF/I, and we speculate that this region is important for receptor binding. Our data support that the interactions between this region and target substrates can be distinct. Fibronectin binding is established to involve charged residues, and neuraminidase treatment did not diminish EAEC binding to fibronectin (Fig. 6A). However, AAF-dependent adherence to mucin and human cells was neuraminidase-sensitive (Fig. 6A and 7A). Two uncharged residues, T67 and N78, were identified as important for substrate binding. Adherence of the N78A variant to both mucin and Caco-2 cells remained sensitive to neuraminidase treatment (Fig. 6B and 7B), suggesting that N78 does not mediate specificity for binding to sialic acid. By contrast, adherence of the T67A variant to Caco-2 cells was not sensitive to neuraminidase treatment (Fig. 7B), and adherence of the T67A variant is more variable than wild type to mucin and Caco-2 cells, both with and without neuraminidase treatment (Fig. 6B and 7B). Together, these data lead us to speculate that T67 may participate in sialic acid binding. The T67A modification results in a smaller side chain lacking a hydroxyl group, and it is possible that this change results in increased flexibility of the binding pocket leading to less specific binding to sialic acid. Although our data from experiments with wild-type AafA and neuraminidase treatment suggest that binding to mucin and cells involves a sialylated molecule, it is possible that other features (the protein backbone or other glycosylation patterns) also contribute to binding specificity by AAF. This is an avenue for future investigation.

In this study, we expand upon previous structure-function studies characterizing AAF-fibronectin interactions to include the biologically relevant molecules mucin and sialic acid as well as interactions with human intestinal cells. Our findings highlight two regions of the protein important for receptor binding and identified both charged and uncharged residues that participate in these interactions. Although both mucin and fibronectin are sialylated, the mechanisms of AAF binding to these molecules are distinct. Overall, our data provide insight into the structural features that determine AAF binding to mucin and models of the human gastrointestinal mucosa.

## SUPPLEMENTAL MATERIAL

Fig. S1 iai.00483-24-s0001.pdf Prediction of AafA folding by AlphaFold.

Fig. S2 iai.00483-24-s0002.pdf TEM of wild-type and variant fimbrial structures.

Fig. S3 iai.00483-24-s0003.pdf Sequences used for modeling of AafA dimer.

Fig. S4 iai.00483-24-s0004.pdf Immunoblots used for quantification of AafA protein levels in Fig 1A.

Supplemental data iai.00483-24-s0005.xlsx Data used to generate figures.

Table S1 iai.00483-24-s0006.docx Bacterial strains used.

ASM does not own the copyrights to Supplemental Material that may be linked to, or accessed through, an article. The authors have granted ASM a non-exclusive, world-wide license to publish the Supplemental Material files. Please contact the corresponding author directly for reuse.
