# Supplementary figures and images for: Structural basis of aggregative adherence fimbriae II interactions with sialic acid, mucin, and human intestinal cells
Source: Infect Immun. 2025 Mar 3;93(4):e00483-24. doi: 10.1128/iai.00483-24 (PMC11977319; doi:10.1128/iai.00483-24)

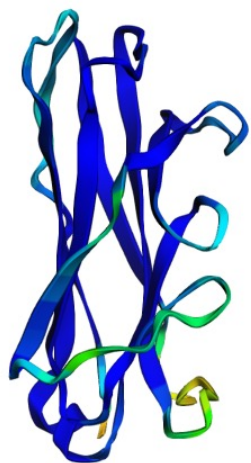

Wild-type

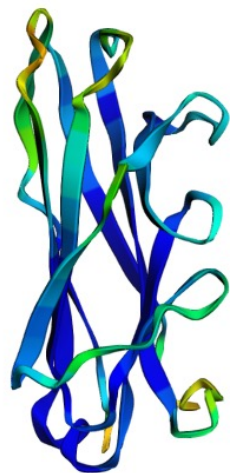

R30A

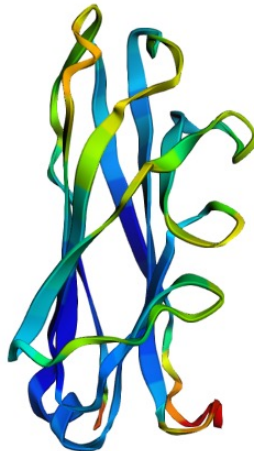

R34A

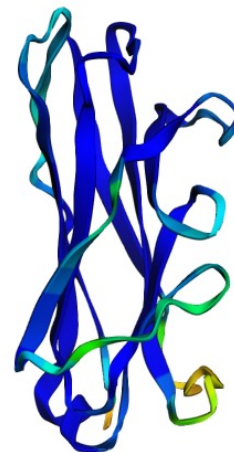

N78A

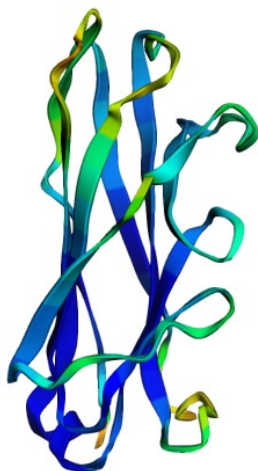

K56A

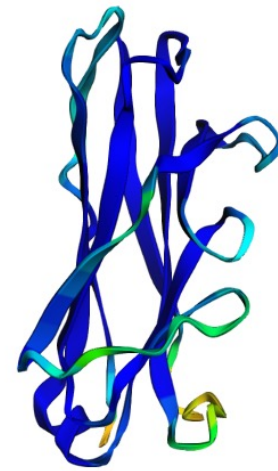

K62A

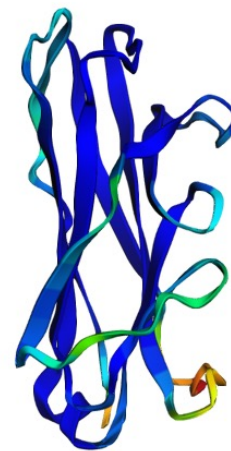

T67A

Supplement: Fig. S1 — Prediction of AafA folding by AlphaFold. [file iai.00483-24-s0001.pdf]
