# Supplementary material for: Structural basis of aggregative adherence fimbriae II interactions with sialic acid, mucin, and human intestinal cells
Source: Infect Immun. 2025 Mar 3;93(4):e00483-24. doi: 10.1128/iai.00483-24 (PMC11977319; doi:10.1128/iai.00483-24)
Supplement: Fig. S2 — TEM of wild-type and variant fimbrial structures. [file iai.00483-24-s0002.pdf]

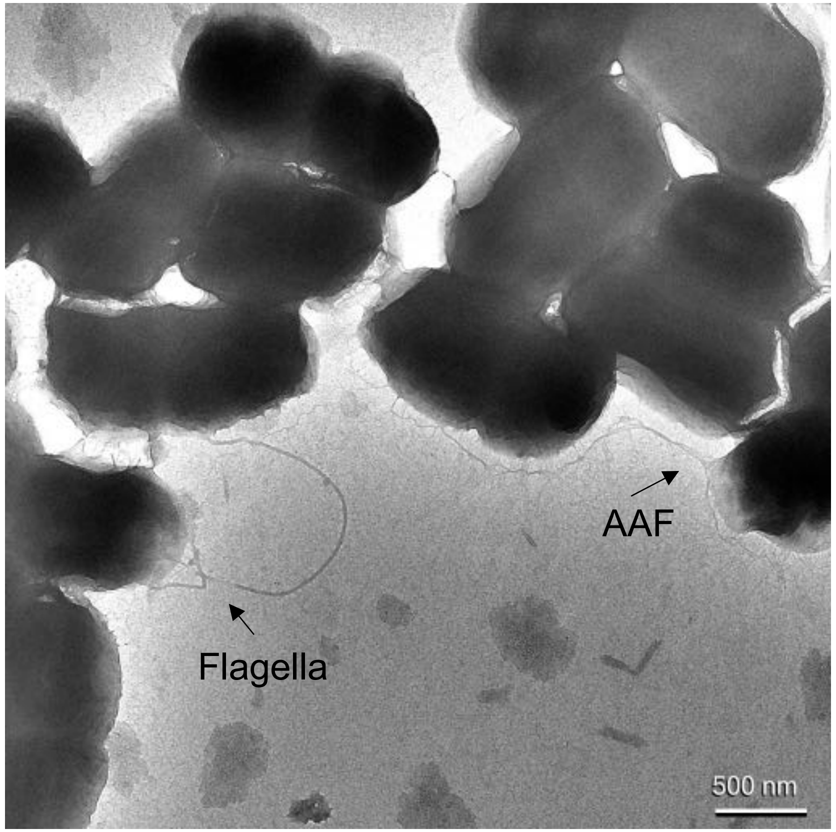

042*aafA*(pBAD*aafDA*)
9,600X

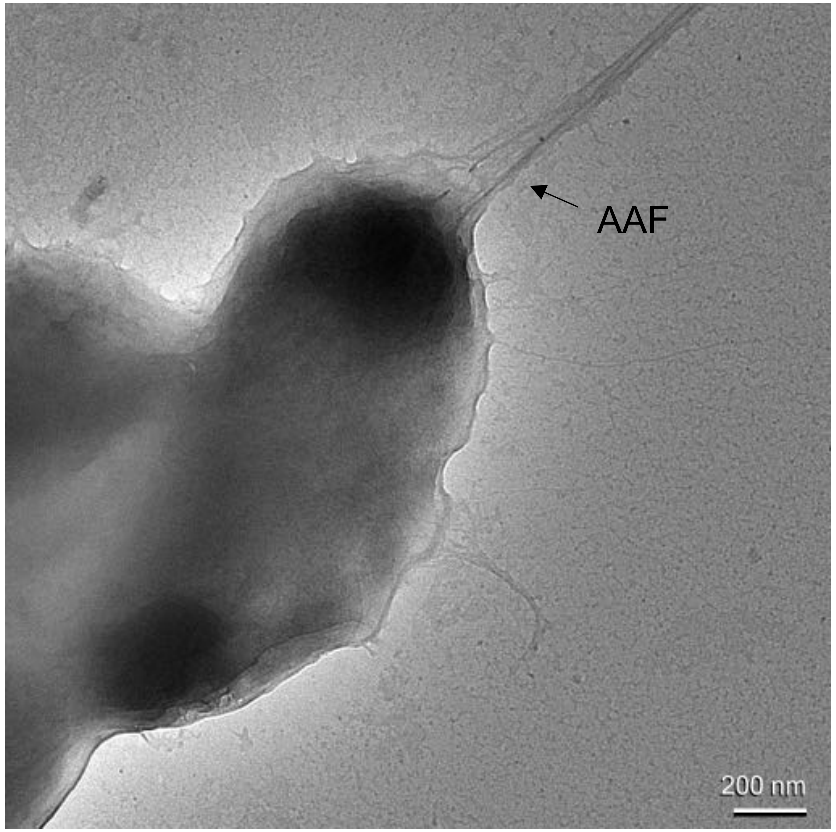

042*aafA*(pBAD*aafDA*)
19,000X

Fig S2. Transmission electron microscopy of 042*aafA*(pBAD*aafDA*) and variants. Bacterial strains were grown statically for 16-18 h in DMEM-HG with 2% arabinose. The bacteria were washed in PBS and then fixed overnight at 4°C in 2.5% glutaraldehyde (Electron Microscopy Sciences). After fixation, the bacteria were washed and diluted to an OD600 of approximately 0.9 in ultrapure water. The bacteria were allowed to adhere to Formvar-coated copper grids, and excess liquid was wicked away with filter paper. The cells were then stained with Uranyless (Electron Microscopy Sciences) and imaged on a 200kV FEI Tecnai F20 operated at 120kV. The microscope is equipped with a 4k x 4k UltraScan CCD camera. AAF are 3-5 nm in diameter, and are flexible/prone to bundling in an Eiffel Tower-like formation. Flagella are wider in diameter with a characteristic hook or curve.

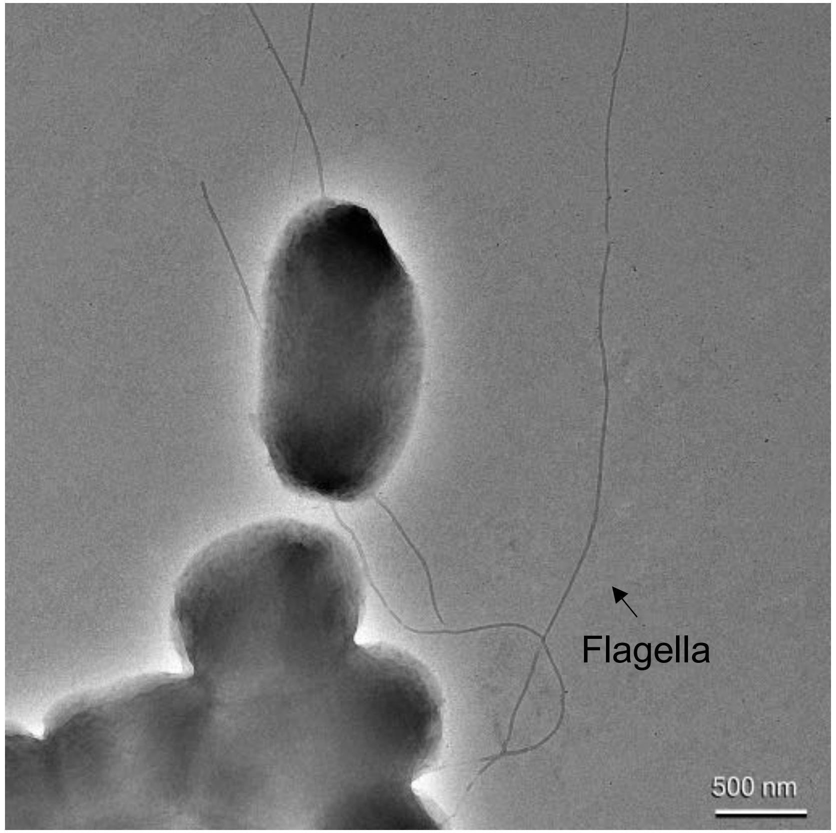

042*aafA* (negative control)
9,600X

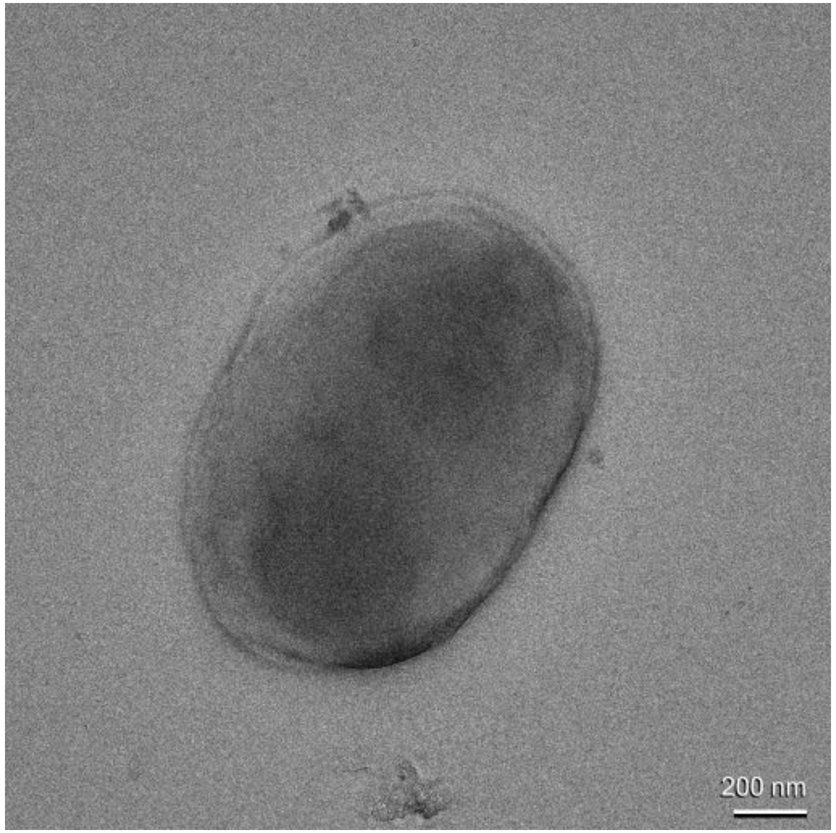

042*aafA* (negative control)
9,600X

Fig S2. Transmission electron microscopy of *aafA* pBAD*aafDA* and variants. Bacterial strains were grown statically for 16-18 h in DMEM-HG with 2% arabinose. The bacteria were washed in PBS and then fixed overnight at 4°C in 2.5% glutaraldehyde (Electron Microscopy Sciences). After fixation, the bacteria were washed and diluted to an OD600 of approximately 0.9 in ultrapure water. The bacteria were allowed to adhere to Formvar-coated copper grids, and excess liquid was wicked away with filter paper. The cells were then stained with Uranyless (Electron Microscopy Sciences) and imaged on a 200kV FEI Tecnai F20 operated at 120kV. The microscope is equipped with a 4k x 4k UltraScan CCD camera. AAF are 3-5 nm in diameter, and are flexible/prone to bundling in an Eiffel Tower-like formation. Flagella are wider in diameter with a characteristic hook or curve.

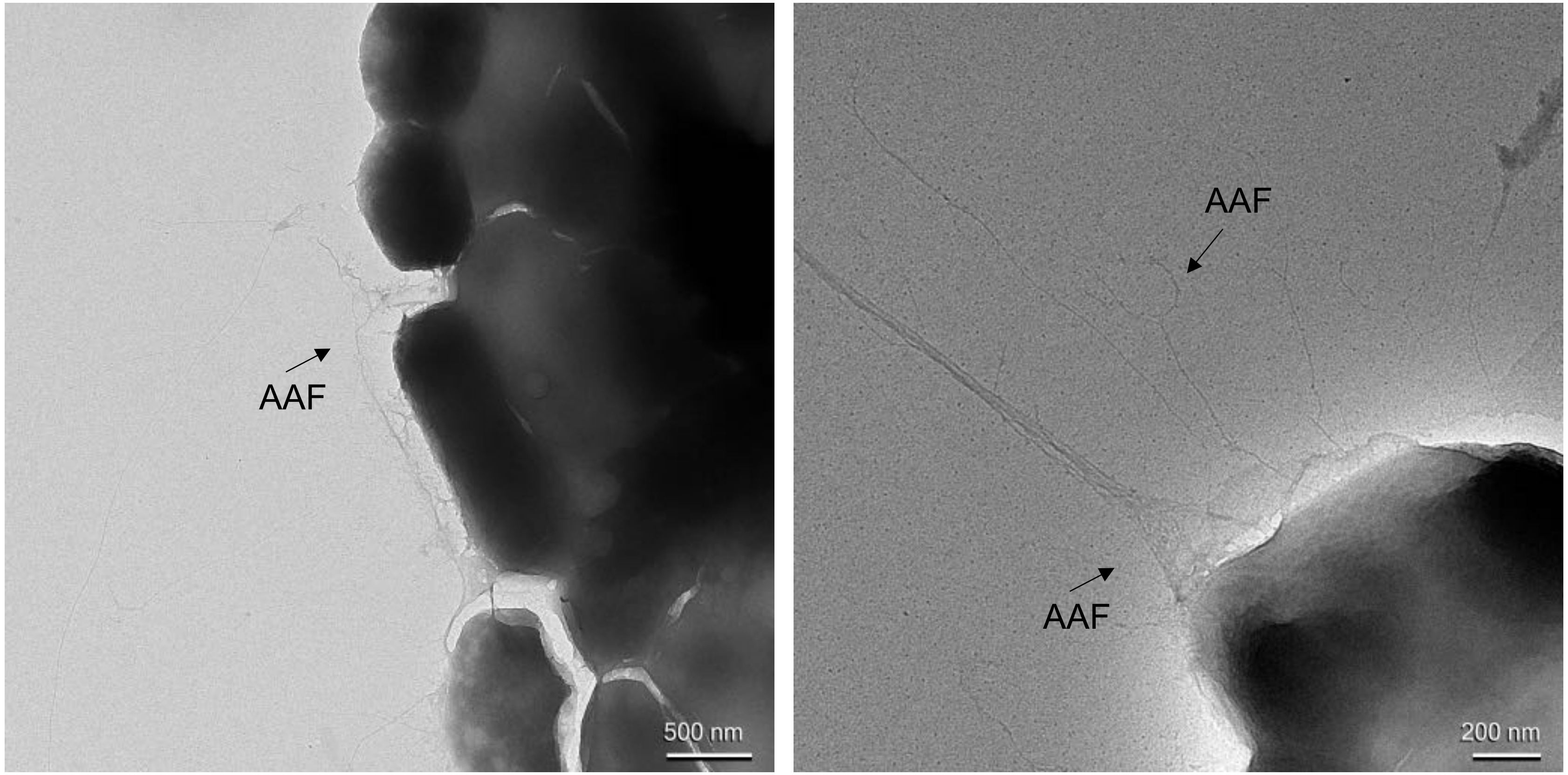

K56A
9,600X

K56A
19,000X

Fig S2. Transmission electron microscopy of *aafA* pBAD*aafDA* and variants. Bacterial strains were grown statically for 16-18 h in DMEM-HG with 2% arabinose. The bacteria were washed in PBS and then fixed overnight at 4°C in 2.5% glutaraldehyde (Electron Microscopy Sciences). After fixation, the bacteria were washed and diluted to an OD600 of approximately 0.9 in ultrapure water. The bacteria were allowed to adhere to Formvar-coated copper grids, and excess liquid was wicked away with filter paper. The cells were then stained with Uranyless (Electron Microscopy Sciences) and imaged on a 200kV FEI Tecnai F20 operated at 120kV. The microscope is equipped with a 4k x 4k UltraScan CCD camera. AAF are 3-5 nm in diameter, and are flexible/prone to bundling in an Eiffel Tower-like formation. Flagella are wider in diameter with a characteristic hook or curve.

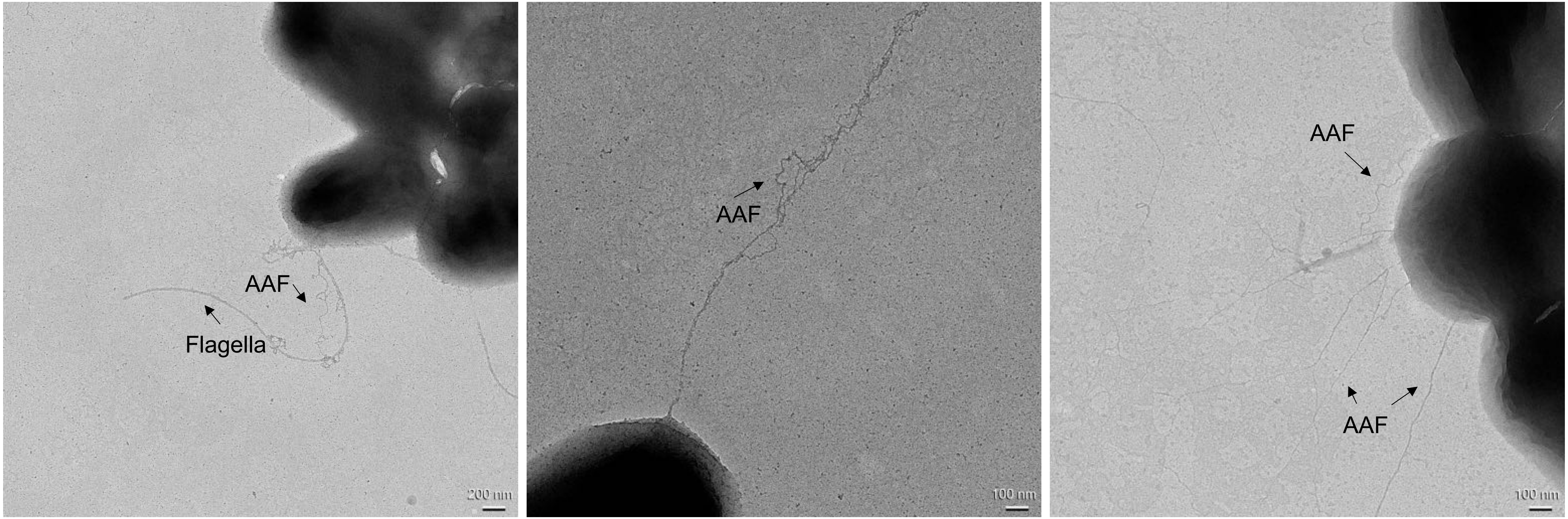

R34A
9,600X

R34A
19,000X

R34A
19,000X

Fig S2. Transmission electron microscopy of *aafA* pBAD*aafDA* and variants. Bacterial strains were grown statically for 16-18 h in DMEM-HG with 2% arabinose. The bacteria were washed in PBS and then fixed overnight at 4°C in 2.5% glutaraldehyde (Electron Microscopy Sciences). After fixation, the bacteria were washed and diluted to an OD600 of approximately 0.9 in ultrapure water. The bacteria were allowed to adhere to Formvar-coated copper grids, and excess liquid was wicked away with filter paper. The cells were then stained with Uranyless (Electron Microscopy Sciences) and imaged on a 200kV FEI Tecnai F20 operated at 120kV. The microscope is equipped with a 4k x 4k UltraScan CCD camera. AAF are 3-5 nm in diameter, and are flexible/prone to bundling in an Eiffel Tower-like formation. Flagella are wider in diameter with a characteristic hook or curve.
