# Supplementary material for: Structural basis of aggregative adherence fimbriae II interactions with sialic acid, mucin, and human intestinal cells
Source: Infect Immun. 2025 Mar 3;93(4):e00483-24. doi: 10.1128/iai.00483-24 (PMC11977319; doi:10.1128/iai.00483-24)
Supplement: Fig. S3 — Sequences used for modeling of AafA dimer. [file iai.00483-24-s0003.pdf]

Key:
Yellow = native c terminus

Green = signal sequence

Red text = flexible "linker" added to dsc form

Purple = N-terminal extension

Native AafA allele from EAEC strain 042 (GenBank: BAJ79294.1)
MKKIRMFVIATLLSSGAAINATAVAKTATSTITVVNNCDITITPATNRDVNVDRSANIDLSFTIRQPQRCADAGMRIKAWGEGNHGQLLIKPQGGNKSAGF
TLASPRFSYIPNNPTNIMNGFVLTNPGVYQLGMQGSITPAMPLRPGIYEVVLNAELVTN

AafA-dsc used for NMR structure (PDB ID: 2MPV)

NFCDITITPATNRDVNVDRSANIDLSFTIRQPQRCADAGMRIKAWGEANHGQLLIKPQGGNKSAGFTLASPRFSYIPNNPANIMNGFVLTNPGVYQLGM
QGSITPAIPLRPGLYEVVLNAELVTNDNKQNATAVAKTATSTITVV

Molecule 1: native without N-terminal extension, signal sequence is cleaved
NNCDITITPATNRDVNVDRSANIDLSFTIRQPQRCADAGMRIKAWGEGNHGQLLIKPQGGNKSAGFTLASPRFSYIPNNPTNIMNGFVLTNPGVYQLGM
QGSITPAMPLRPGIYEVVLNAELVTN

Molecule 2: DSC form with "extra" N-terminal extension, signal sequence is cleaved

NATAVAKTATSTITVVNFCDITITPATNRDVNVDRSANIDLSFTIRQPQRCADAGMRIKAWGEANHGQLLIKPQGGNKSAGFTLASPRFSYIPNNPANIMN
GFVLTNPGVYQLGMQGSITPAIPLRPGLYEVVLNAELVTNDNKQNATAVAKTATSTITVV

Fig S3. Sequences used for modeling of AafA dimer.
