# Supplementary material for: Structural basis of aggregative adherence fimbriae II interactions with sialic acid, mucin, and human intestinal cells
Source: Infect Immun. 2025 Mar 3;93(4):e00483-24. doi: 10.1128/iai.00483-24 (PMC11977319; doi:10.1128/iai.00483-24)
Supplement: Fig. S4 — Immunoblots used for quantification of AafA protein levels in Fig 1A. [file iai.00483-24-s0004.pdf]

Blot 1

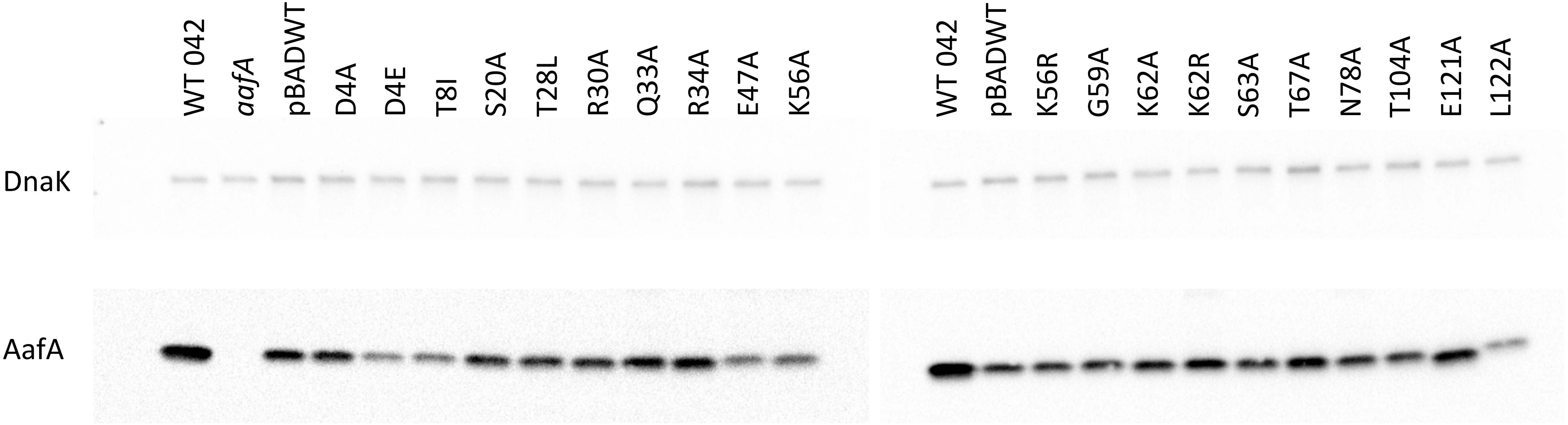


**Fig S4. Immunoblots used for densitometry analyses.** Bacterial strains were grown statically for 20 hours in DMEM-high glucose with 2% arabinose and 100ug/ml carbenicillin. Cultures were standardized by $OD_{600}$ before Western blot analyses. For whole cell lysates, cells were collected by centrifugation, washed, and lysed under denaturing conditions. Samples were probed with rabbit polyclonal AafA antisera or anti-DnaK (Thermo Fisher) and visualized by chemiluminescence (BioRad).

Blot 2

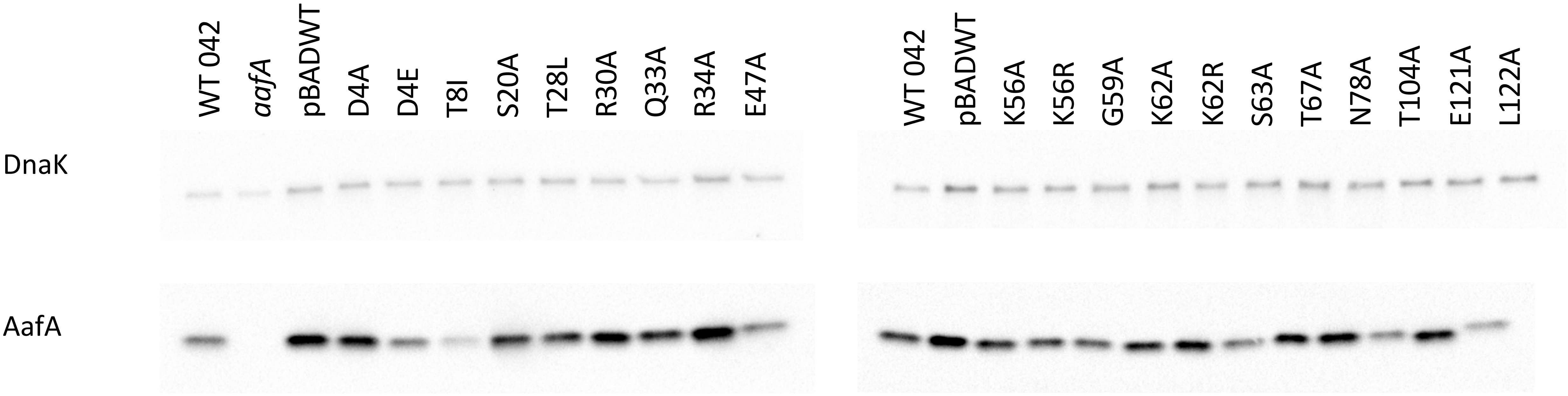


**Fig S4. Immunoblots used for densitometry analyses.** Bacterial strains were grown statically for 20 hours in DMEM-high glucose with 2% arabinose and 100ug/ml carbenicillin. Cultures were standardized by $OD_{600}$ before Western blot analyses. For whole cell lysates, cells were collected by centrifugation, washed, and lysed under denaturing conditions. Samples were probed with rabbit polyclonal AafA antisera or anti-DnaK (Thermo Fisher) and visualized by chemiluminescence (BioRad).

Blot 3

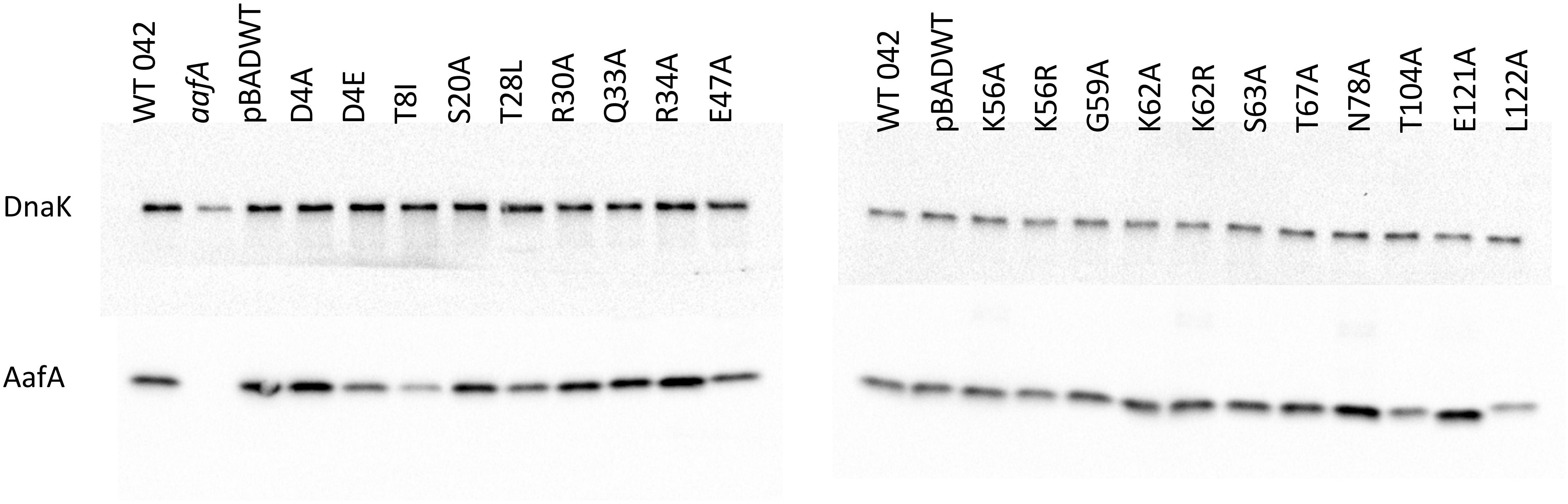


**Fig S4. Immunoblots used for densitometry analyses.** Bacterial strains were grown statically for 20 hours in DMEM-high glucose with 2% arabinose and 100ug/ml carbenicillin. Cultures were standardized by $OD_{600}$ before Western blot analyses. For whole cell lysates, cells were collected by centrifugation, washed, and lysed under denaturing conditions. Samples were probed with rabbit polyclonal AafA antisera or anti-DnaK (Thermo Fisher) and visualized by chemiluminescence (BioRad).
