# Supplementary material for: Structural basis of aggregative adherence fimbriae II interactions with sialic acid, mucin, and human intestinal cells
Source: Infect Immun. 2025 Mar 3;93(4):e00483-24. doi: 10.1128/iai.00483-24 (PMC11977319; doi:10.1128/iai.00483-24)
Supplement: Table S1 — Bacterial strains used. [file iai.00483-24-s0006.docx]

**Supplemental Table 1. Bacterial strains used in this study.** Sequences for AafA single-amino acid substitutions listed with affected residue colored red.

| Strain | Description |
| --- | --- |
| 042 | Prototype EAEC strain isolated from a child with diarrhea in Lima, Peru (1) |
| 042*aafA* | Kanamycin-insertion mutant of *aafA* in 042 created using lambda red linear recombination method (2), also referred to as EAEC*aafA*::*km*-*sacB* (3) |
| *aafA* repair | Strain with *aafA* restored to normal location within pAA2 plasmid (4) |
| 042*aafA(*pBAD*aafDA)* | 042 *aafa* harboring pBAD30 with *aafDA* under inducible expression with arabinose (5)  Contains wild-type sequence of AafA: MKKIRMFVIATLLSSGAAINATAVAKTATSTITVVNNCDITITPATNRDVNVDRSANIDLSFTIRQPQRCADAGMRIKAWGEGNHGQLLIKPQGGNKSAGFTLASPRFSYIPNNPTNIMNGFVLTNPGVYQLGMQGSITPAMPLRPGIYEVVLNAELVTN |
| 042*aafA(*pBAD*aafDA)* D4A | MKKIRMFVIATLLSSGAAINATAVAKTATSTITVVNNC**A**ITITPATNRDVNVDRSANIDLSFTIRQPQRCADAGMRIKAWGEGNHGQLLIKPQGGNKSAGFTLASPRFSYIPNNPTNIMNGFVLTNPGVYQLGMQGSITPAMPLRPGIYEVVLNAELVTN, described in (5) |
| 042*aafA(*pBAD*aafDA)* D4E | MKKIRMFVIATLLSSGAAINATAVAKTATSTITVVNNC**E**ITITPATNRDVNVDRSANIDLSFTIRQPQRCADAGMRIKAWGEGNHGQLLIKPQGGNKSAGFTLASPRFSYIPNNPTNIMNGFVLTNPGVYQLGMQGSITPAMPLRPGIYEVVLNAELVTN |
| 042*aafA(*pBAD*aafDA)* T8I | MKKIRMFVIATLLSSGAAINATAVAKTATSTITVVNNCDITI**I**PATNRDVNVDRSANIDLSFTIRQPQRCADAGMRIKAWGEGNHGQLLIKPQGGNKSAGFTLASPRFSYIPNNPTNIMNGFVLTNPGVYQLGMQGSITPAMPLRPGIYEVVLNAELVTN, described in (5) |
| 042*aafA(*pBAD*aafDA)* S20A | MKKIRMFVIATLLSSGAAINATAVAKTATSTITVVNNCDITITPATNRDVNVDR**A**ANIDLSFTIRQPQRCADAGMRIKAWGEGNHGQLLIKPQGGNKSAGFTLASPRFSYIPNNPTNIMNGFVLTNPGVYQLGMQGSITPAMPLRPGIYEVVLNAELVTN, described in (5) |
| 042*aafA(*pBAD*aafDA)* T28L | MKKIRMFVIATLLSSGAAINATAVAKTATSTITVVNNCDITITPATNRDVNVDRSANIDLSF**L**IRQPQRCADAGMRIKAWGEGNHGQLLIKPQGGNKSAGFTLASPRFSYIPNNPTNIMNGFVLTNPGVYQLGMQGSITPAMPLRPGIYEVVLNAELVTN, described in (5) |
| 042*aafA(*pBAD*aafDA)* R30A | MKKIRMFVIATLLSSGAAINATAVAKTATSTITVVNNCDITITPATNRDVNVDRSANIDLSFTI**A**QPQRCADAGMRIKAWGEGNHGQLLIKPQGGNKSAGFTLASPRFSYIPNNPTNIMNGFVLTNPGVYQLGMQGSITPAMPLRPGIYEVVLNAELVTN, described in (5) |
| 042*aafA(*pBAD*aafDA)* Q33A | MKKIRMFVIATLLSSGAAINATAVAKTATSTITVVNNCDITITPATNRDVNVDRSANIDLSFTIRQP**A**RCADAGMRIKAWGEGNHGQLLIKPQGGNKSAGFTLASPRFSYIPNNPTNIMNGFVLTNPGVYQLGMQGSITPAMPLRPGIYEVVLNAELVTN, described in (5) |
| 042*aafA(*pBAD*aafDA)* R34A | MKKIRMFVIATLLSSGAAINATAVAKTATSTITVVNNCDITITPATNRDVNVDRSANIDLSFTIRQPQ**A**CADAGMRIKAWGEGNHGQLLIKPQGGNKSAGFTLASPRFSYIPNNPTNIMNGFVLTNPGVYQLGMQGSITPAMPLRPGIYEVVLNAELVTN, described in (5) |
| 042*aafA(*pBAD*aafDA)* E47A | MKKIRMFVIATLLSSGAAINATAVAKTATSTITVVNNCDITITPATNRDVNVDRSANIDLSFTIRQPQRCADAGMRIKAWG**A**GNHGQLLIKPQGGNKSAGFTLASPRFSYIPNNPTNIMNGFVLTNPGVYQLGMQGSITPAMPLRPGIYEVVLNAELVTN, described in (5) |
| 042*aafA(*pBAD*aafDA)* K56A | MKKIRMFVIATLLSSGAAINATAVAKTATSTITVVNNCDITITPATNRDVNVDRSANIDLSFTIRQPQRCADAGMRIKAWGEGNHGQLLI**A**PQGGNKSAGFTLASPRFSYIPNNPTNIMNGFVLTNPGVYQLGMQGSITPAMPLRPGIYEVVLNAELVTN, described in (5) |
| 042*aafA(*pBAD*aafDA)* K56R | MKKIRMFVIATLLSSGAAINATAVAKTATSTITVVNNCDITITPATNRDVNVDRSANIDLSFTIRQPQRCADAGMRIKAWGEGNHGQLLI**R**PQGGNKSAGFTLASPRFSYIPNNPTNIMNGFVLTNPGVYQLGMQGSITPAMPLRPGIYEVVLNAELVTN, described in (5) |
| 042*aafA(*pBAD*aafDA)* G59A | MKKIRMFVIATLLSSGAAINATAVAKTATSTITVVNNCDITITPATNRDVNVDRSANIDLSFTIRQPQRCADAGMRIKAWGEGNHGQLLIKPQ**A**GNKSAGFTLASPRFSYIPNNPTNIMNGFVLTNPGVYQLGMQGSITPAMPLRPGIYEVVLNAELVTN, described in (5) |
| 042*aafA(*pBAD*aafDA)* K62A | MKKIRMFVIATLLSSGAAINATAVAKTATSTITVVNNCDITITPATNRDVNVDRSANIDLSFTIRQPQRCADAGMRIKAWGEGNHGQLLIKPQGGN**A**SAGFTLASPRFSYIPNNPTNIMNGFVLTNPGVYQLGMQGSITPAMPLRPGIYEVVLNAELVTN, described in (5) |
| 042*aafA(*pBAD*aafDA)* K62R | MKKIRMFVIATLLSSGAAINATAVAKTATSTITVVNNCDITITPATNRDVNVDRSANIDLSFTIRQPQRCADAGMRIKAWGEGNHGQLLIKPQGGN**R**SAGFTLASPRFSYIPNNPTNIMNGFVLTNPGVYQLGMQGSITPAMPLRPGIYEVVLNAELVTN, described in (5) |
| 042*aafA(*pBAD*aafDA)* S63A | MKKIRMFVIATLLSSGAAINATAVAKTATSTITVVNNCDITITPATNRDVNVDRSANIDLSFTIRQPQRCADAGMRIKAWGEGNHGQLLIKPQGGNK**A**AGFTLASPRFSYIPNNPTNIMNGFVLTNPGVYQLGMQGSITPAMPLRPGIYEVVLNAELVTN, described in (5) |
| 042*aafA(*pBAD*aafDA)* T67A | MKKIRMFVIATLLSSGAAINATAVAKTATSTITVVNNCDITITPATNRDVNVDRSANIDLSFTIRQPQRCADAGMRIKAWGEGNHGQLLIKPQGGNKSAGF**A**LASPRFSYIPNNPTNIMNGFVLTNPGVYQLGMQGSITPAMPLRPGIYEVVLNAELVTN, described in (5) |
| 042*aafA(*pBAD*aafDA)* N78A | MKKIRMFVIATLLSSGAAINATAVAKTATSTITVVNNCDITITPATNRDVNVDRSANIDLSFTIRQPQRCADAGMRIKAWGEGNHGQLLIKPQGGNKSAGFTLASPRFSYIP**A**NPTNIMNGFVLTNPGVYQLGMQGSITPAMPLRPGIYEVVLNAELVTN, described in (5) |
| 042*aafA(*pBAD*aafDA)* T104A | MKKIRMFVIATLLSSGAAINATAVAKTATSTITVVNNCDITITPATNRDVNVDRSANIDLSFTIRQPQRCADAGMRIKAWGEGNHGQLLIKPQGGNKSAGFTLASPRFSYIPNNPTNIMNGFVLTNPGVYQLGMQGSI**A**PAMPLRPGIYEVVLNAELVTN, described in (5) |
| 042*aafA(*pBAD*aafDA)* E121A | MKKIRMFVIATLLSSGAAINATAVAKTATSTITVVNNCDITITPATNRDVNVDRSANIDLSFTIRQPQRCADAGMRIKAWGEGNHGQLLIKPQGGNKSAGFTLASPRFSYIPNNPTNIMNGFVLTNPGVYQLGMQGSITPAMPLRPGIYEVVLNA**A**LVTN, described in (5) |
| 042*aafA(*pBAD*aafDA)* L122A | MKKIRMFVIATLLSSGAAINATAVAKTATSTITVVNNCDITITPATNRDVNVDRSANIDLSFTIRQPQRCADAGMRIKAWGEGNHGQLLIKPQGGNKSAGFTLASPRFSYIPNNPTNIMNGFVLTNPGVYQLGMQGSITPAMPLRPGIYEVVLNAE**A**VTN, described in (5) |

References

1. Nataro JP, Deng Y, Cookson S, Cravioto A, Savarino SJ, Guers LD, Levine MM, Tacket CO. 1995. Heterogeneity of enteroaggregative Escherichia coli virulence demonstrated in volunteers. The Journal of Infectious Diseases 171:465–468.

2. Datsenko KA, Wanner BL. 2000. One-step inactivation of chromosomal genes in Escherichia coli K-12 using PCR products. Proc Natl Acad Sci USA 97:6640–6645.

3. Izquierdo M, Navarro-Garcia F, Nava-Acosta R, Nataro JP, Ruiz-Perez F, Farfan MJ. 2014. Identification of cell surface-exposed proteins involved in the fimbria-mediated adherence of enteroaggregative Escherichia coli to intestinal cells. Infect Immun 82:1719–1724.

4. Gonyar LA, Smith RM, Giron JA, Zachos NC, Ruiz-Perez F, Nataro JP. 2020. Aggregative Adherence Fimbriae II of Enteroaggregative Escherichia coli Are Required for Adherence and Barrier Disruption during Infection of Human Colonoids. Infect Immun 88.

5. Berry AA, Yang Y, Pakharukova N, Garnett JA, Lee W, Cota E, Marchant J, Roy S, Tuittila M, Liu B, Inman KG, Ruiz-Perez F, Mandomando I, Nataro JP, Zavialov AV, Matthews S. 2014. Structural insight into host recognition by aggregative adherence fimbriae of enteroaggregative Escherichia coli. PLoS Pathog 10:e1004404.
